# Phase Coupling of a Circadian Neuropeptide With Rest/Activity Rhythms Detected Using a Membrane-Tethered Spider Toxin

**DOI:** 10.1371/journal.pbio.0060273

**Published:** 2008-11-04

**Authors:** Ying Wu, Guan Cao, Beth Pavlicek, Xuan Luo, Michael N Nitabach

**Affiliations:** Cellular and Molecular Physiology, Yale School of Medicine, New Haven, Connecticut, United States of America; University of Geneva, Switzerland

## Abstract

*Drosophila* clock neurons are self-sustaining cellular oscillators that rely on negative transcriptional feedback to keep circadian time. Proper regulation of organismal rhythms of physiology and behavior requires coordination of the oscillations of individual clock neurons within the circadian control network. Over the last decade, it has become clear that a key mechanism for intercellular communication in the circadian network is signaling between a subset of clock neurons that secrete the neuropeptide pigment dispersing factor (PDF) and clock neurons that possess its G protein-coupled receptor (PDFR). Furthermore, the specific hypothesis has been proposed that PDF-secreting clock neurons entrain the phase of organismal rhythms, and the cellular oscillations of other clock neurons, via the temporal patterning of secreted PDF signals. In order to test this hypothesis, we have devised a novel technique for altering the phase relationship between circadian transcriptional feedback oscillation and PDF secretion by using an ion channel–directed spider toxin to modify voltage-gated Na^+^ channel inactivation in vivo. This technique relies on the previously reported “tethered-toxin” technology for cell-autonomous modulation of ionic conductances via heterologous expression of subtype-specific peptide ion channel toxins as chimeric fusion proteins tethered to the plasma membrane with a glycosylphosphatidylinositol (GPI) anchor. We demonstrate for the first time, to our knowledge, the utility of the tethered-toxin technology in a transgenic animal, validating four different tethered spider toxin ion channel modifiers for use in *Drosophila*. Focusing on one of these toxins, we show that GPI-tethered Australian funnel-web spider toxin δ-ACTX-Hv1a inhibits Drosophila para voltage-gated Na^+^ channel inactivation when coexpressed in *Xenopus* oocytes. Transgenic expression of membrane-tethered δ-ACTX-Hv1a in vivo in the PDF-secreting subset of clock neurons induces rhythmic action potential bursts and depolarized plateau potentials. These in vitro and in vivo electrophysiological effects of membrane-tethered δ-ACTX-Hv1a are consistent with the effects of soluble δ-ACTX-Hv1a purified from venom on Na^+^ channel physiological and biophysical properties in cockroach neurons. Membrane-tethered δ-ACTX-Hv1a expression in the PDF-secreting subset of clock neurons induces an approximately 4-h phase advance of the rhythm of PDF accumulation in their terminals relative to both the phase of the day:night cycle and the phase of the circadian transcriptional feedback loops. As a consequence, the morning anticipatory peak of locomotor activity preceding dawn, which has been shown to be driven by the clocks of the PDF-secreting subset of clock neurons, phase advances coordinately with the phase of the PDF rhythm of the PDF-secreting clock neurons, rather than maintaining its phase relationship with the day:night cycle and circadian transcriptional feedback loops. These results (1) validate the tethered-toxin technology for cell-autonomous modulation of ion channel biophysical properties in vivo in transgenic *Drosophila*, (2) demonstrate that the kinetics of *para* Na^+^ channel inactivation is a key parameter for determining the phase relationship between circadian transcriptional feedback oscillation and PDF secretion, and (3) provide experimental support for the hypothesis that PDF-secreting clock neurons entrain the phase of organismal rhythms via the temporal patterning of secreted PDF signals.

## Author Summary

The regulation of the daily fluctuations that characterize an organism's physiology and behavior requires coordination of the cellular oscillations of individual “clock” neurons within the circadian control network. Clock neurons that secrete a neuropeptide called pigment dispersing factor (PDF) calibrate, or entrain, both the phase of organismal rhythms and the cellular oscillations of other clock neurons. In this study, we tested the hypothesis that phase of PDF secretion rhythms entrains phase of non-PDF neurons and locomotor rhythms using the tethered- toxin technique (which affixes toxins to the cell membrane) to express ion channel–specific peptide toxins in PDF neurons. A particular toxin inhibits inactivation of the Drosophila para sodium (Na^+^) channel. Inhibition of Na^+^ channel inactivation in PDF neurons of transgenic flies induces phase advance of PDF rhythm, and correlated phase advance of lights-on anticipatory locomotor activity, suggesting that phase of morning activity is determined by phase of PDF oscillation. Therefore, voltage-gated Na^+^ channels of *Drosophila* clock neurons play a key role in determining the phase relationship between circadian transcriptional feedback oscillation and PDF secretion, and PDF-secreting clock neurons entrain the phase of organismal rhythms via the temporal patterning of secreted PDF signals.

## Abstract 3

Cell-autonomous inhibition of*Drosophila para* Na^+^ channel inactivation using a membrane-tethered spider toxin phase shifts circadian neuropeptide output from cellular oscillation, and the phase of morning anticipatory activity is determined by this phase-shifted neuropeptide output.

## Introduction

*Drosophila* clock neurons are self-sustaining cellular oscillators that rely on negative transcriptional feedback and cellular signaling pathways to keep circadian time. PERIOD (PER) and TIMELESS (TIM) clock proteins constitute the core of a negative transcriptional feedback loop, in which these clock proteins inhibit transcription of the genes that encode them (for review, see [1,2]). The transcription factors Vrille (VRI), par domain protein 1 (PDP1), along with PER and TIM, form two transcriptional feedback loops interconnected through CLOCK/CYCLE-mediated transcriptional activation [3–8]. These feedback loops interact to produce daily rhythms of clock gene mRNA and clock protein accumulation, which are required for rhythmic physiology and behavior (for review, see [2,9–11]). Recent studies have also implicated depolarization-dependent ionic conductances and intracellular Ca^2+^ signals in cellular oscillation in the fly [12,13].

Proper regulation of organismal rhythms of physiology and behavior requires coordination of the cellular oscillations of the individual clock neurons within the circadian control network [14–21]. A key role has been suggested for pigment dispersing factor (PDF) secretion by the lateral ventral subset of clock neurons in this coordination, as *pdf^01^*-null mutant flies, ventral lateral neuron (LN_V_)-ablated flies, or LN_V_-electrically silenced flies all exhibit disrupted free-running locomotor rhythms [12,16,17]. It has been proposed that cyclic release of PDF from the dorsomedial terminals of small PDF neurons (sLN_V_) provides an important circadian signal to PDF-sensitive downstream targets [18,20–22]. In the absence of PDF, *pdf^01^*-null flies exhibit dispersed and desynchronized phase relationships of independent cellular oscillators [21]. Hyperexcitation of LN_V_s via expression of a slowly inactivating bacterial Na^+^ channel disrupts cyclic release of PDF, and results in desynchrony among subsets of clock neurons and complex behavioral rhythms [18]. These findings suggest that the PDF-secreting LN_V_s synchronize independent clock oscillators via clock-regulated PDF secretion. The PDF receptor (PDFR) G protein-coupled receptor appears to be functionally active in many clock neurons, and PDFR mutant flies exhibit disrupted free-running locomotor rhythms similar to *pdf^01^*-null mutant flies [23–26]. These studies further confirm the key role of PDF neuropeptide in circadian control of locomotor activity, indicating the importance of intercellular communication in the circadian network between PDF-secreting LN_V_s and clock neurons that express PDFR (for review, see [11]).

Furthermore, the specific hypothesis has been proposed that PDF-secreting clock neurons entrain both the phase of organismal rhythms and cellular oscillations of other clock neurons [17,27–29]. PDF injected into the cockroach brain resets the phase rather than the period of free-running behavioral rhythms [29], suggesting that PDF mediates a nonphotic entraining signal to the circadian clock. In the absence of PDF in *pdf^01^*-null mutant flies, phase of LN_V_ and non-LN_V_ clock neurons disperses, and these flies exhibit no morning anticipatory peak of locomotor activity and a phase-advanced evening anticipatory peak [17,21]. This suggests that non-LN_V_ clock neurons free-run when not receiving entraining PDF signal from LN_V_s and that PDF-dependent intercellular communication in the circadian control network is required to maintain the proper phase relationship between morning and evening activity peaks. Morning and evening anticipatory peaks are thought to be controlled by “morning” cells (M cells), the PDF-secreting LN_V_s, and “evening” cells (E cells), the dorsal LN_D_s/DN1s, respectively [30,31]. In constant darkness (DD), M cells dominate the clock network and entrain the phase of E cells and organismal rhythms by sending the E cells a daily resetting signal [27]. In constant light, genetically modified E cells can drive organismal locomotor rhythms [28,32,33], in some situations independently of PDF signals [32,33]. The daily resetting signal that M cells send to E cells has been hypothesized to be PDF itself [27].

In order to test the hypothesis that phase of PDF secretion rhythms entrains phase of E cells and locomotor rhythms, we have devised a novel technique for altering the phase relationship between circadian transcriptional feedback oscillation and rhythmic PDF secretion by LN_V_s using an ion channel–specific spider toxin to modify voltage-gated *para* Na^+^ channel inactivation in vivo. This technique relies on the previously reported “tethered-toxin” technology for cell-autonomous modulation of ionic conductances via heterologous expression of subtype-specific peptide ion channel toxins as chimeric fusion proteins tethered to the plasma membrane with a glycosylphosphatidylinositol (GPI) anchor [34]. Membrane-tethered toxins act only on ion channels present in the membrane of the cell that is expressing the toxin, and not on identical ion channels present on neighboring cells that do not express the toxin. In this study, we demonstrate for the first time, to our knowledge, the utility of the tethered-toxin technology in a transgenic animal, validating four different tethered spider toxin ion channel modifiers for use in *Drosophila*. Focusing on one of these toxins, GPI-tethered Australian funnel-web spider toxin δ-ACTX-Hv1a, which inhibits Drosophila para voltage-gated Na^+^ channel inactivation when coexpressed in *Xenopus* oocytes, we (1) validate the tethered-toxin technology for cell-autonomous modulation of ion channel biophysical properties in vivo in transgenic *Drosophila*, (2) demonstrate that the kinetics of *para* Na^+^ channel inactivation is a key parameter for determining the phase relationship between circadian transcriptional feedback oscillation and PDF secretion, and (3) provide experimental support for the hypothesis that PDF-secreting clock neurons entrain organismal and cellular rhythms via the temporal patterning of secreted PDF signals.

## Results

### Screen for Membrane-Tethered Ion Channel Toxins Active in *Drosophila* Circadian Clock Neurons

It has recently been demonstrated that peptide ion channel toxins from venomous predators can be heterologously expressed as fusion proteins covalently tethered to GPI anchors inserted in the extracellular leaflet of the plasma membrane [34]. Membrane-tethered toxins are expressed as chimeric fusion protein comprising (from N to C termini) a secretory signal sequence, peptide toxin sequence, glycine-asparagine repeat hydrophilic linker, and GPI targeting sequence. Membrane-tethered toxins are targeted to the secretory pathway, where the signal sequence is cleaved, and the GPI targeting sequence is substituted by covalent bond to GPI, thus retaining the toxin on the surface of the cell in which it is expressed. The modulatory effects of these tethered toxins are limited to ion channels on the surface of the cells in which the toxin is expressed, and do not influence channels on neighboring cells not expressing membrane-tethered toxin [34].

**Figure 1:**
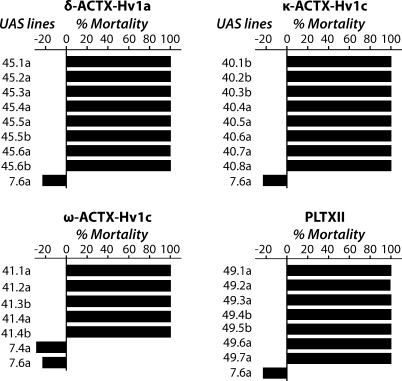
Pan-Neuronal Expression of Membrane-Tethered Spider Toxins Induces Lethality in Transgenic *Drosophila* Flies homozygous for an *elav*-*GAL4* transgene were mated to flies heterozygous for the indicated *UAS-toxin* transgenes and a phenotypically marked balancer chromosome, such that one half of the progeny receive both *elav*-*GAL4* and *UAS-toxin* transgenes, while the other half of the progeny receive *elav*-*GAL4* and the balancer chromosome. Lethality was assessed as percent relative mortality, which is 100% if there are no viable adult flies with both transgenes, and 0% if there are the same number of viable adult flies with both transgenes as with *elav*-*GAL4* and the balancer. Negative mortality reflects a fitness disadvantage of progeny carrying a balancer chromosome relative to progeny carrying a chromosome with a *UAS-toxin* transgene insertion. The code numbers refer to independent chromosomal insertions of the indicated *UAS-toxin* transgenes. Code numbers 7.4a and 7.6a indicate two independent insertions of a membrane-tethered μO-MrVIA *UAS* transgene, which encodes a vertebrate Na^+^ channel blocker with no activity against *Drosophila* ion channels. Pan-neuronal expression of membrane-tethered μO-MrVIA has no effect on viability of flies. However, pan-neuronal expression of either one of the four indicated spider toxins induces complete embryonic/larval lethality for all independent *UAS-toxin* chromosomal insertions tested. *n* > 50 animals for each cross.

We selected 19 different peptide ion channel toxins from the venoms of cone snails, scorpions, bees, and spiders (Table 1) for testing in vivo in membrane-tethered form in the nervous system of transgenic Drosophila melanogaster. Of the 19 membrane-tethered toxins tested, four of them cause complete embryonic/larval lethality when expressed pan-neuronally using an *elav-GAL4* driver (Figure 1), indicating activity against functionally essential fly ion channel subtypes. Three of these four membrane-tethered toxins are derived from the venom of the Australian funnel web spider Hadronyche versuta. δ-ACTX-Hv1a inhibits inactivation of Na^+^ channels in isolated cockroach giant axon and dorsal unpaired median neurons and rat dorsal root ganglion neurons, and induces spontaneous repetitive action potential (AP) firing accompanied by plateau potentials [35]. κ-ACTX-Hv1c has highly conserved structural features common to multiple K^+^ channel blockers and has been shown to target *Drosophila* K^+^ channels [36,37]. ω-ACTX-Hv1c is a blocker of mid/low- and high-voltage–activated insect Ca^2+^ channels [38,39]. PLTXII, a toxin from Plectreurys tristis spider venom, inhibits neurotransmission at the *Drosophila* neuromuscular synapse and targets the presynaptic Ca^2+^ channel encoded by the *cacophony* gene [40,41]. As shown in Figure 1, pan-neuronal expression of any of these four toxins induces embryonic/early-larval lethality when expressed from any one of multiple independent chromosomal insertions. In contrast, μO-MrVIA, a blocker of vertebrate voltage-gated Na^+^ channels from the venom of a fish-eating cone snail [42], has no effect when expressed pan-neuronally (Figure 1). Lack of effect of μO-MrVIA when expressed pan-neuronally indicates absence of activity against fly Na^+^ channels, at least in its tethered form. We thus utilize tethered μO-MrVIA as a negative control for all experiments. The fact that the only four toxins showing signs of bioactivity when expressed in membrane-tethered form in the *Drosophila* nervous system are derived from spiders, and not from ocean-dwelling cone snails, makes sense in light of the natural prey species of spiders and cone snails.

**Table 1:**
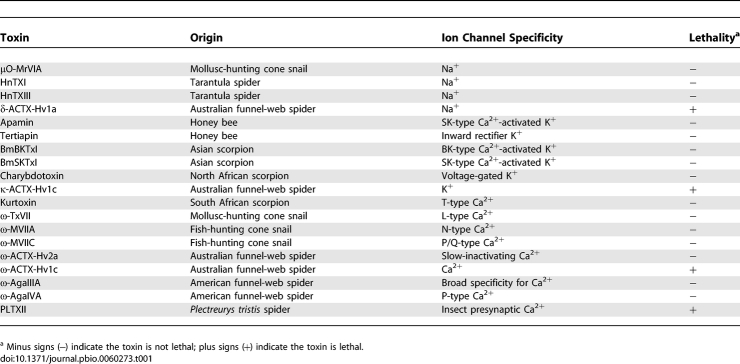
Peptide Ion Channel Toxins Expressed Pan-Neuronally in Membrane-Tethered Form

**Figure 2:**
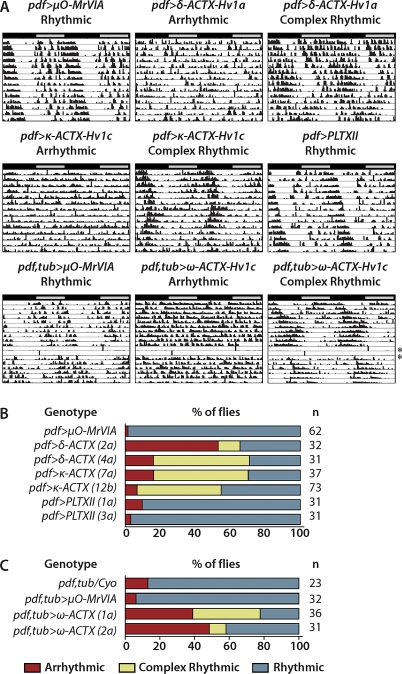
Behavioral Arrhythmicity or Complex Rhythmicity Induced by Membrane-Tethered Toxin Expression (A) Double-plotted locomotor actograms of representative individual male progeny of the indicated genotypes are shown, spanning 14 d in DD after release from diurnal 12-h:12-h LD entraining conditions. The bar above each actogram indicates subjective day (gray) and subjective night (black). *pdf>μO-MrVIA* or *pdf,tub>μO-MrVIA* control flies expressing μO-MrVIA in PDF neurons (LN_V_s) exhibit a single statistically significant free-running rhythm of locomotor activity. *tub* indicates the presence of a *tub-GAL80^ts^* transgene. *tub-GAL80^ts^* is a temperature-sensitive mutant form of the GAL80 transcriptional repressor. At the permissive temperature of 18 °C, GAL80^ts^ is active at repressing GAL4-mediated transcription, but at the restrictive temperature of 30 °C, it is inactive. Therefore, GAL4-mediated toxin expression can be temporally activated by shifting adult *pdf,tub>μO-MrVIA* or *pdf,tub>ω-ACXT-Hv1c* flies from 18 °C to 30 °C. In contrast, *pdf>δ-ACTX-Hv1a* flies or *pdf>κ-ACTX-Hv1c* flies expressing toxin in the LN_V_s frequently exhibit behavioral arrhythmicity or complex rhythmicity of locomotor activity. PLTXII expression in the LN_V_s has no behavioral effect on locomotor activity. *pdf,tub>ω-ACXT-Hv1c* flies expressing ω-ACXT-Hv1c driven by *pdf-GAL4* and *tub-GAL80^ts^* were allowed to develop to adult stage at 18 °C and tested for behavior at 30 °C, and exhibit behavioral arrhythmicity or complex rhythmicity in comparison to control *pdf,tub>μO-MrVIA* flies. The asterisk (*) in the bottom row of actograms indicates 2 d during which the locomotor monitoring system was inoperative. (B) A summary of the percentages of flies exhibiting arrhythmic (red), complex rhythmic (yellow), or single rhythmic (blue) locomotor activity assayed over the first 14 d in DD. The difference in proportion of behavioral phenotypes between control *pdf>μO-MrVIA* flies and experimental *pdf>δ-ACTX-Hv1a* or *pdf>κ-ACTX-Hv1a* flies expressing toxin from different insertions are statistically significant by χ^2^ test; for each of the χ^2^ values, *p* < 0.0001. *pdf>PLTXII* flies were not statistically distinguishable from control. *n*, number of flies tested. (C) A summary of the percentages of *pdf,tub>ω-ACXT-Hv1c* flies exhibiting behavioral phenotypes at 30 °C. The difference in proportion of behavioral phenotypes between control *pdf,tub>μO-MrVIA* flies and experimental *pdf,tub>ω-ACTX-Hv1c* from different insertions are statistically significant by χ^2^ test; for each of the χ^2^ values, *p* < 0.0001. *n*, number of flies tested.

To determine whether these four tethered spider toxins are active against ion channels important for circadian pacemaker neuron function, we expressed membrane-tethered toxins solely in the LN_V_ subset of approximately 20 clock neurons that secrete PDF and are considered key pacemakers of the clock circuit [12,16,17]. Three of the four potent membrane-tethered toxins substantially disrupt free-running circadian rhythms of locomotor activity when expressed in PDF neurons using a *pdf-GAL4* driver (Figure 2, Table S1). δ-ACTX-Hv1a or κ-ACTX-Hv1c expression in PDF neurons induces arrhythmicity or complex rhythmicity in individual toxin-expressing flies. Complex rhythmicity occurs when an individual fly exhibits multiple rhythms of locomotor activity free-running simultaneously with different periods (Figure 2). In contrast, expression of tethered vertebrate Na^+^ channel blocker μO-MrVIA in PDF neurons has no behavioral effect on locomotor rhythms in comparison either with the background strain used for transgenesis (unpublished data) or with *pdf-GAL4* driver flies (Figure 2, Table S1). Membrane-tethered PLTXII Ca^2+^ channel blocker has no effect on circadian behavior when expressed in PDF neurons (Figure 2). Membrane-tethered ω-ACTX-Hv1c expression in PDF neurons using the *pdf-GAL4* driver, which is active in both PDF-expressing clock neurons as well as in neurosecretory cells in the thoracic ganglion starting in early larval development [8,17], causes death by approximately 1 wk posteclosion, suggesting the possibility of toxin-induced developmental defects. To eliminate such possible developmental effects of toxin expression, we exploited temperature-dependent temporal control of GAL4-driven transgene expression using a temperature-sensitive mutant form of GAL80 transcriptional repressor (GAL80^ts^), which binds to GAL4 and inhibits downstream transcription that would otherwise be activated by GAL4 [43,44]. At 18 °C, GAL80^ts^ dominates GAL4′s activator function and is active at repressing transcription, but at 30 °C, it is inactive. Thus, GAL4-mediated transgene expression can be temporally activated by shifting flies that also express GAL80^ts^ from 18 °C to 30 °C. Flies expressing membrane-tethered ω-ACTX-Hv1c with both *pdf-GAL4* and *tub-GAL80^ts^* were allowed to develop to adulthood at 18 °C, and then were shifted to 30 °C to induce membrane-tethered ω-ACTX-Hv1c expression. These flies have a normal lifespan, but exhibit circadian rhythm defects of arrhythmicity or complex rhythmicity, as shown in Figure 2 and Table S1. These results indicate the presence of functionally important ion channels sensitive to κ-ACTX-Hv1c, ω-ACTX-Hv1c, and δ-ACTX-Hv1a in PDF-secreting LN_V_ clock neurons.

### Membrane-Tethered δ-ACTX-Hv1a Dose-Dependently Inhibits Inactivation of Drosophila para Na^+^ Channel When Coexpressed in *Xenopus* Oocytes

**Figure 3:**
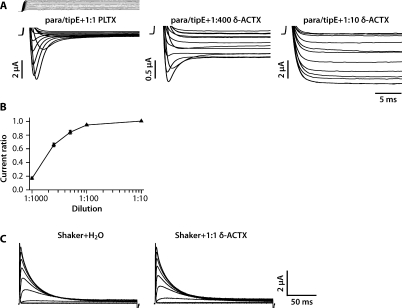
Membrane-Tethered δ-ACTX-Hv1a Abolishes Inactivation of Drosophila para Voltage-Gated Na^+^ Channel *Xenopus* oocytes were injected with same ratio of para/tipE cRNA by weight (1.66/1.32 ng/nl), and inward Na^+^ currents were recorded in response to step depolarizations from holding potential of −90 mV to a series of test potentials from −70 mV to 40 mV in 10 mV increments. (A) Representative recordings from oocytes injected with cRNA encoding para/tipE and membrane-tethered PLTXII Ca^2+^ channel toxin or membrane-tethered δ-ACTX-Hv1a Na^+^ channel toxin. Para/tipE conducts inward Na^+^ current exhibiting fast activation and inactivation. Membrane-tethered δ-ACTX-Hv1a totally abolishes inactivation of the current when injected at 1:10, and partially abolishes inactivation at 1:400 dilution. Large transient capacitative currents at the beginning and end of the voltage steps were cropped. Current ratio refers to the ratio of steady state current to peak current for the depolarizing step to −30 mV. (B) The curve shows the relationship between membrane-tethered toxin cRNA dilution ratio and current ratio (*I*_ss_/*I_P_*). Membrane-tethered δ-ACTX -Hv1a inhibits the inactivation of *para* Na^+^ channel in a dose-dependent manner. (C) Representative recordings from oocytes injected with cRNA encoding *Drosophila Shaker* K^+^ channel and membrane-tethered δ-ACTX-Hv1a or H_2_O. Shaker conducts outward K^+^ current exhibiting fast activation and inactivation. Membrane-tethered δ-ACTX-Hv1a has no effect on Shaker inactivation.

δ-ACTX-Hv1a has been shown to inhibit inactivation of insect and mammalian voltage-gated Na^+^ currents [35]. The predominant *Drosophila* voltage-gated Na^+^ channel is encoded by the *para* gene [45,46]. Functional expression of *para* in *Xenopus* oocytes is enhanced by coexpression of the tipE accessory subunit [47,48]. We coexpressed membrane-tethered δ-ACTX-Hv1a with para/tipE to examine the effect of the tethered toxin on *para* voltage-gated Na^+^ currents. Uninjected oocytes showed no voltage-gated inward currents (unpublished data). The inward Na^+^ current recorded from oocytes injected with para/tipE complementary RNA (cRNA) exhibits voltage-dependent activation, with rapid and complete inactivation occurring within several milliseconds (Figure 3A). Coexpression of tethered δ-ACTX-Hv1a dose-dependently inhibits the inactivation of *para* Na^+^ current. We used the ratio of steady-state current to peak current during 40 ms of depolarization to −30 mV to compare the extent of inhibition by membrane-tethered δ-ACTX-Hv1a of *para* Na^+^ current inactivation. Coinjection of para/tipE and either H_2_O or tethered PLTXII Ca^2+^ channel toxin cRNAs (1:1) induces inward Na^+^ current with the current ratio of 0.023 ± 0.015 (*n* = 4) or 0.029 ± 0.004 (*n* = 16), respectively (mean ± standard error of the mean [SEM]). A low concentration of 1:1,000 tethered δ-ACTX-Hv1a cRNA significantly decreased the inactivation of Na^+^ current to a current ratio of 0.169 ± 0.018 (*n* = 21, *p* < 0.001). A high concentration of toxin (1:10) completely abolishes inactivation of Na^+^ current resulting in a current ratio of 1.005 ± 0.003 (*n* = 8, *p* < 0.001; Figure 3). In contrast, tethered δ-ACTX-Hv1a has no effect on kinetics and amplitude of *Drosophila Shaker* K^+^ current, which also exhibits fast activation and inactivation (Figure 3C), indicating retained specificity of membrane-tethered δ-ACTX-Hv1a for inhibiting inactivation of voltage-gated Na^+^ channels.

### Functional Expression of Membrane-Tethered δ-ACTX-Hv1a in PDF-Secreting LN_V_ Pacemaker Neurons Dramatically Alters Spontaneous Electrical Activity

**Figure 4:**
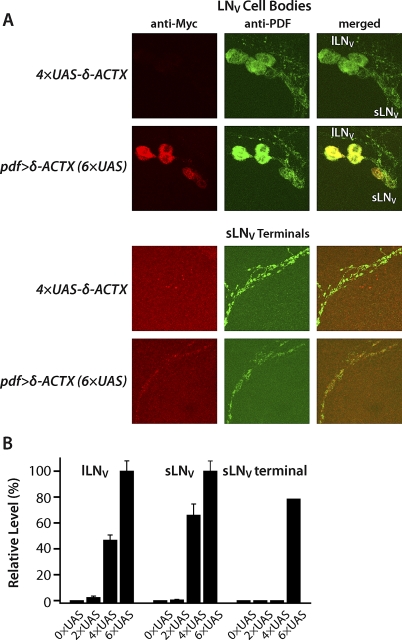
Immunofluorescence Detection of Membrane-Tethered δ-ACTX-Hv1a in PDF-Secreting LN_V_ Clock Neurons Adult brains of *pdf>δ-ACTX-Hv1a* flies possessing the indicated number of *UAS* transgenes and the *pdf-GAL4* transgene, or flies only possessing the indicated number of *UAS-δ-ACTX-Hv1a* transgenes, were processed for immunofluorescence with anti-Myc and anti-PDF antibodies to visualize both Myc epitope-tagged membrane-tethered δ-ACTX-Hv1a and PDF neuropeptide. (A) *pdf>δ-ACTX-Hv1a* (6×UAS) flies exhibit red anti-Myc immunofluorescence in the cell bodies of small LN_V_s (sLN_V_s) and large LN_V_s (lLN_V_s), sLN_V_ dorsomedial terminals, and lLN_V_ projections to the opposite optic lobe (not shown in this figure). Anti-Myc immunofluorescence colocalizes with green anti-PDF in the cell bodies of PDF neurons. Red anti-Myc immunofluorescence exhibits punctate staining throughout the sLN_V_ terminals. (B) Bar graph demonstrates the dose-dependent expression of δ-ACTX-Hv1a in the lLN_V_s and sLN_V_s (*p* < 0.001; ANOVA Tukey-Kramer multiple comparisons). The intensity level (mean ± SEM) of anti-Myc labeling in the lLN_V_s and sLN_V_s is normalized to the intensity level in flies with six copies of *UAS-δ-ACTX-Hv1a* expressed with *pdf-GAL4* driver. Bar graph for sLN_V_ terminals represents percentage of brain hemispheres exhibiting anti-Myc staining in the sLN_V_ terminals.

To confirm membrane targeting of membrane-tethered δ-ACTX-Hv1a in vivo, we examined the expression of Myc-tagged δ-ACTX-Hv1a in PDF-secreting clock neurons. The ten-amino acid Myc epitope tag is located in the middle of the glycine-asparagine repeat hydrophilic linker domain. Brains of flies with two, four, or six copies of *UAS-δ-ACTX-Hv1a* and *pdf-GAL4* were double immunostained with anti-Myc and anti-PDF fluorescence, with parental 4×*UAS-δ-ACTX-Hv1a* flies as negative control for anti-Myc staining. As shown in Figure 4, control *UAS-δ-ACTX-Hv1a* flies show no anti-Myc immunofluorescence in the LN_V_ cell bodies or terminals. Anti-PDF labels the cell bodies of PDF neurons (LN_V_s) and their processes, including dorsomedial terminals of sLN_V_s (Figure 4A) and projection of large LN_V_s (lLN_V_s) to the contralateral optic lobe through posterior optic tract (unpublished data). Flies expressing δ-ACTX-Hv1a in LN_V_s exhibit red anti-Myc immunofluorescence colocalized with anti-PDF green fluorescence in the cell bodies of LN_V_s. δ-ACTX-Hv1a expression dose-dependently increases anti-Myc immunofluorescence detected in the cell bodies of both lLN_V_s and sLN_V_s, as well as sLN_V_ dorsomedial terminals (Figure 4). Red anti-Myc immunofluorescence is barely detectable in cell bodies of lLN_V_ in *pdf>*2×*UAS-δ-ACTX-Hv1a* flies, and not at all in the sLN_V_s. Introduction of four or six copies of *UAS-δ-ACTX-Hv1a* transgene significantly increases anti-Myc immunofluorescence detected in the cell bodies of both lLN_V_s and sLN_V_s (*p* < 0.001, Figure 4). Anti-Myc immunofluorescence in sLN_V_ dorsomedial terminals is not seen in brain hemispheres of *pdf>2×UAS-δ-ACTX-Hv1a* or *pdf>4×UAS-δ-ACTX-Hv1a* 4 flies. In *pdf>6×UAS-δ-ACTX-Hv1a* flies, 11 out of 16 hemispheres (68.8%) exhibit detectable anti-Myc immunofluorescence in the sLN_V_ dorsomedial terminals (Figure 4B). Projection of lLN_V_s to the opposite optic lobes through the posterior optic tract is also visualized with anti-Myc immunofluorescence in these brains (unpublished data). These results establish that membrane-tethered δ-ACTX-Hv1a is expressed in LN_V_ clock neurons and is transported throughout their neuronal arbors. Membrane-tethered ω-ACTX-Hv1c, a Ca^2+^ channel blocker, expression in LN_V_s is shown in Figure S1. High-resolution confocal images of the dorsomedial terminals of sLN_V_s expressing membrane-tethered ω-ACTX-Hv1c demonstrate green anti-Myc fluorescence signal surrounding “puncta” of red anti-PDF signal. This is consistent with targeting of ω-ACTX-Hv1c to the plasma membrane of the terminals that encloses regions of concentration of PDF-containing dense-core vesicles. These results indicate that membrane-tethered spider toxins are expressed by LN_V_ clock neurons and targeted to the plasma membrane where they can interact with their target ion channels.

**Figure 5:**
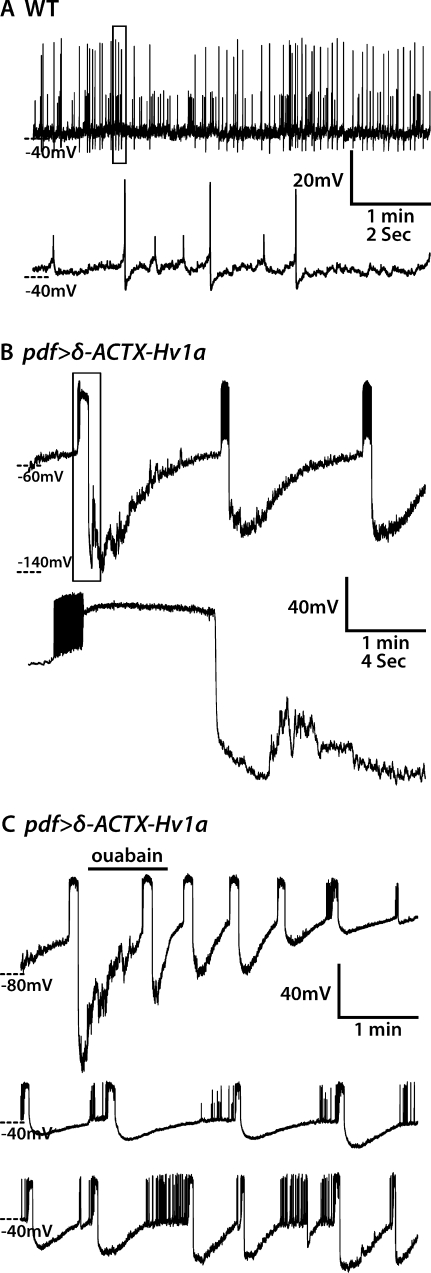
Membrane Activity of Large PDF Neurons (lLN_V_s) Is Dramatically Altered by Membrane-Tethered δ-ACTX-Hv1a Expression (A) Representative whole-cell recording of WT lLN_V_ is shown. The upper panel is a typical 5-min recording, and the lower panel depicts a magnified view of the outlined 10-s segment of the 5-min trace. The resting membrane potential of the WT lLN_V_ is relatively stable (∼−40mV), and two different sizes of spontaneous action potentials are observed, as previously reported [49]. (B) Representative 5-min whole-cell recording of lLN_V_ expressing membrane-tethered δ-ACTX-Hv1a from two copies of *UAS- δ-ACTX-Hv1a* transgene and *pdf-GAL4* driver is shown in the upper panel. The lower panel depicts a magnified view of the outlined 20-s segment of the 5-min trace. Immediately following an action potential burst, the membrane potential remains at a −15mV depolarized plateau for approximately 8 s before a rapid massive hyperpolarization that is slowly recovered from. (C) Continuous 15-min recording of another lLN_V_ expressing membrane-tethered δ-ACTX-Hv1a from two copies of *UAS- δ-ACTX-Hv1a*. 0.1 mM ouabain was applied for 1 min as indicated by the horizontal bar. Ouabain dramatically reduces the magnitude of postplateau hyperpolarization, suggesting its origin in Na^+^/K^+^-ATPase pump current. The continued increase in ouabain effect following washout is likely due to slow tissue diffusion of the hydrophobic drug.

In order to confirm functional expression of membrane-tethered δ-ACTX-Hv1a, we performed whole-cell electrophysiological recordings on lLN_V_ clock neurons expressing δ-ACTX-Hv1a. Previous studies indicate that lLN_V_ RMP and AP firing rate is regulated by the circadian clock to encode time of day [49,50]. In 12-h:12-h light:dark (LD) conditions, lLN_V_ RMP varies within the range of −40 mV to −70 mV. For any given cell steady state, lLN_V_ RMP remains relatively stable, albeit frequently with approximately 5−10-mV membrane potential oscillation. In the representative example shown in Figure 5A, this WT lLN_V_ RMP is relatively stable at −40 mV. Membrane-tethered δ-ACTX-Hv1a–expressing lLN_V_ RMP exhibits dramatically different membrane-activity from WT lLN_V_s. A representative example is shown in Figure 5B. Membrane-tethered δ-ACTX-Hv1a–expressing lLN_V_ membrane potential oscillates in a very wide range from approximately −140 to approximately 0 mV. Membrane-tethered δ-ACTX-Hv1a–expressing lLN_V_ RMP becomes gradually depolarized, and spontaneous AP firing rate increases as the membrane potential level increases. When the RMP reaches approximately −40 mV, a burst of 5−30 APs lasting approximately 0.2–0.5 s occurs. After the burst, the membrane potential remains at a depolarized plateau (−15−0 mV) for 2−7 s. Following the plateau, membrane potential repolarizes rapidly down to approximately −140 mV within 2 s. Following this huge hyperpolarization, membrane potential gradually depolarizes to eventually initiate another cycle of this unique behavior. We have recorded from 53 membrane-tethered δ-ACTX-Hv1a–expressing lLN_V_s, and 36 of them exhibit this basic cyclic temporal pattern of membrane potential. However, the exact frequency of these cycles, the duration of AP bursts and depolarized plateau, and the number of APs during the AP burst, exhibit substantial variation from cell to cell, even when expressing the same number of copies of *δ-ACTX-Hv1a* transgene. lLN_V_s expressing different numbers of *δ-ACTX-Hv1a* transgene do not show consistent differences in their membrane potential dynamics. Since membrane-tethered δ-ACTX-Hv1a inhibits inactivation of *para* voltage-gated Na^+^ channels (Figure 3), the AP burst and prolonged depolarized plateau in membrane-tethered δ-ACTX-Hv1a–expressing lLN_V_s most likely involve sustained opening of *para* channels, substantial Na^+^ influx, and increased intracellular [Na^+^]. We thus tested the hypothesis that the massive postplateau hyperpolarization well below the reversal potential for K^+^ is induced by electrogenic Na^+^/K^+^-ATPase pump currents expelling all that intracellular Na^+^, by blocking those currents with ouabain [51]. In the representative example shown in Figure 5C, the application of 0.1 mM ouabain for 1 min substantially reduces the massive postplateau hyperpolarizations in membrane-tethered δ-ACTX-Hv1a–expressing lLN_V_s, and this effect is at least partially reversible. These results suggest that Na^+^/K^+^-ATPase underlies the postplateau hyperpolarization.

### LN_V_ Expression of Membrane-Tethered δ-ACTX-Hv1a Disrupts Free-Running Rhythms of Locomotor Activity

**Figure 6:**
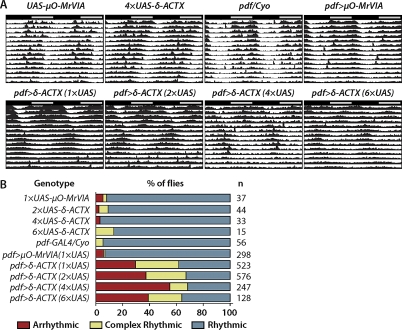
PDF-Secreting LN_V_ Clock Neuron Expression of Membrane-Tethered δ-ACTX-Hv1a Disrupts Free-Running Behavioral Rhythms Flies expressing membrane-tethered μO-MrVIA or δ-ACTX-Hv1a from indicated numbers of copies of *UAS* transgenes using *pdf-GAL4* driver, as well as indicated nonexpressing parental *UAS* and *pdf-GAL4* flies, were entrained in 12-h:12-h LD conditions before release into DD. Double-plotted normalized actograms depict averaged locomotor activity for indicated genotypes of flies from a representative experiment. Bar graph shows proportions of flies with the indicated behavioral phenotypes as assessed by Lomb-Scargle periodogram analysis of all pooled experiments. Control flies expressing membrane-tethered μO-MrVIA or parental transgenic lines not expressing any membrane-tethered peptide exhibit rhythmic locomotor activity with a single free-running rhythm. In contrast, most flies expressing membrane-tethered δ-ACTX-Hv1a exhibit arrhythmicity or complex rhythmicity. The averaged actograms reveal this complex rhythmicity as multiple superimposed free-running rhythms of different periods. (A) The averaged actograms of all flies (including rhythmic and arrhythmic flies) of the indicated genotypes from a representative experiment are shown. *pdf>μO-MrVIA* flies exhibit locomotor rhythm with a single period of approximately 24.4 h. In contrast, *pdf>δ-ACTX-Hv1a* flies exhibit complex rhythmicity with simultaneously superimposed rhythms with periods of approximately 25 h and approximately 22 h. (B) A summary of the percentages of flies exhibiting arrhythmic (red), complex rhythmic (yellow) and single rhythmic (blue) locomotor activity assayed over the first 14 d in DD. The difference in proportion of behavioral phenotypes between each of the control flies, either *pdf>μO-MrVIA* or *UAS-toxin* strain or *pdf-GAL4* driver, and experimental *pdf>δ-ACTX-Hv1a* flies are each statistically significant by χ^2^ test; for each of the χ^2^ values, *p* < 0.0001. *n*, number of flies tested.

To determine whether these physiological modifications of LN_V_s induced by expression of membrane-tethered δ-ACTX-Hv1a affect the function of the circadian control network, we examined free-running locomotor activity patterns of flies expressing either membrane-tethered δ-ACTX-Hv1a or μO-MrVIA specifically in the PDF-expressing LN_V_s using *pdf-GAL4* driver. Control *pdf>μO-MrVIA* flies expressing membrane-tethered μO-MrVIA exhibit rhythmic free-running circadian behavior of locomotor activity with a single statistically significant period of approximately 24.4 h (Figure 6A), of 298 μO-MrVIA–expressing flies tested, 93% of them exhibited a single statistically significant periodogram peak (Figure 6, Table S1). In contrast, substantial numbers of experimental flies expressing membrane-tethered δ-ACTX-Hv1a in the LN_V_s exhibit arrhythmicity or complex rhythmicity (i.e., multiple superimposed free-running circadian rhythms of locomotor activity with different periods: one of approximately 25 h and one of approximately 22 h). Free-running behavioral effects induced by membrane-tethered δ-ACTX-Hv1a expression in the LN_V_s either with single independent chromosomal insertion, or with multiple chromosomal insertions are relatively similar. The remaining rhythmic δ-ACTX-Hv1a–expressing flies exhibit single-period free-running circadian rhythms with period corresponding to either the long or short period of complex rhythmicity, suggesting a similar effect on the circadian control network in these flies, albeit with a different behavioral manifestation (unpublished data). Arrhythmicity or complex rhythmicity is only rarely observed in control *pdf>μO-MrVIA* flies, which almost always exhibit single free-running rhythms (Figure 6, Table S1). The differences in proportion of behavioral phenotypes between membrane-tethered δ-ACTX-Hv1a–expressing flies and control (either membrane-tethered μO-MrVIA–expressing flies or parental *UAS* flies) were all statistically significant (χ^2^; *p* < 0.001).

To exclude a role for developmental effects of membrane-tethered δ-ACTX-Hv1a expression in inducing these behavioral phenotypes, we exploited the GAL80^ts^ system for temporal control of GAL4-driven transgene expression. Flies possessing *UAS-δ-ACTX-Hv1a*, *pdf-GAL4*, and *tub-GAL80^ts^* transgenes were allowed to develop at 18 °C from egg to adult in the absence of δ-ACTX-Hv1a expression, and then shifted to 30 °C to induce membrane-tethered δ-ACTX-Hv1a expression. Experimental *pdf,tub>δ-ACTX-Hv1a* flies exhibit arrhythmicity or complex rhythmicity at 30 °C similar to *pdf>δ-ACTX-Hv1a* flies (Figure S2), indicating that the behavioral phenotype caused by membrane-tethered δ-ACTX-Hv1a expression in the LN_V_s is not due to developmental effects. The percentage of arrhythmic or complex rhythmic flies expressing δ-ACTX-Hv1a from two or four copies of *UAS-δ-ACTX-Hv1a* transgene is significantly greater than control *pdf>μO-MrVIA* or *UAS-toxin* flies (χ^2^; *p* < 0.0001). The exact manifestation of complex rhythmicity in *pdf,tub>δ-ACTX-Hv1a* flies is somewhat different than in *pdf>δ-ACTX-Hv1a* flies. The latter tend to simultaneously manifest strong short- and long-period rhythms immediately after transfer from 12-h:12-h LD into DD, while the former tend to initially manifest mainly a long-period rhythm and then, after about a week in DD, transition to predominately just a short-period rhythm, as seen in published studies of complex rhythmic flies [20]. The most likely interpretation of this particular form of complex rhythmicity at the cellular level is—as in the case in which strong short- and long-period rhythms manifest themselves simultaneously—that there are independently free-running subsets of clock neurons running with short and long periods, with the difference that their respective ability to gate locomotor activity waxes and wanes reciprocally over time in DD. These results indicate that the physiological changes induced by LN_V_ expression of membrane-tethered δ-ACTX-Hv1a interfere with the ability of the circadian control network to drive coherent free-running behavioral rhythms in constant darkness.

### Membrane-Tethered δ-ACTX-Hv1a Expression in LN_V_ Clock Neurons Phase Advances Both PDF Cycling in Dorsomedial sLN_V_ Nerve Terminals and Morning Anticipatory Locomotor Activity

**Figure 7:**
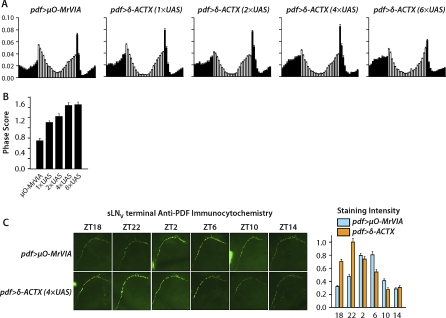
Membrane-Tethered δ-ACTX-Hv1a Expression in PDF-Secreting LN_V_s Induces Phase Advance of Both Morning Anticipatory Locomotor Activity and PDF Accumulation in the Dorsomedial Terminals of sLN_V_s Flies with *pdf-GAL4* and either *UAS- δ-ACTX-Hv1a* (4×UAS) or *UAS-μO-MrVIA* transgenes were entrained for at least 4 d in 12-h:12-h LD conditions and then brains were processed for anti-PDF immunofluorescence. Green anti-PDF signal indicates cytoplasmic PDF accumulation in sLN_V_ dorsomedial terminals. (A) Average activity histograms indicate relative levels of locomotor activity versus time for flies of the indicated genotypes over 5 d in LD. Each bar represents 30-min cumulative activity, with white and black bars indicating the day and night phases. Flies expressing membrane-tethered δ-ACTX-Hv1a exhibit phase advanced lights-on anticipation as revealed by the change in shape of the envelope of relative activity in the 3 h prior to lights-on from concave to convex with increasing membrane-tethered δ-ACTX-Hv1a expression. (B) Bar graph shows computed phase scores (mean ± SEM) for lights-on anticipation, defined as relative cumulative activity in the 3 h just before lights-on divided by the 3 h just after lights-on. Indicated genotypes represent flies expressing either membrane-tethered μO-MrVIA or δ-ACTX-Hv1a with one, two, four or six copies of UAS. Membrane-tethered δ-ACTX-Hv1a expression dose-dependently phase advances morning anticipation of lights-on. The difference between control *pdf>μO-MrVIA* and each of the *pdf> δ-ACTX-Hv1a* genotypes is statistically significant by ANOVA with Tukey-Kramer multiple comparison (*p* < 0.001). (C) Bar graphs show normalized integrated anti-PDF staining intensity (mean ± SEM). Representative immunofluorescence images of sLN_V_ dorsomedial terminals from flies of the indicated genotypes at the indicated time points are shown. Control flies expressing membrane-tethered μO-MrVIA exhibit peak PDF accumulation at ZT2–ZT6 and trough at ZT14–ZT18 (*p* < 0.001). In contrast, membrane-tethered δ-ACTX-Hv1a–expressing flies exhibit peak PDF accumulation at ZT22 and trough at ZT10–ZT14 (*p* < 0.001), representing an approximately 4-h phase advance of the rhythm of PDF accumulation. The differences among different genotypes at different circadian times were compared with ANOVA and Tukey-Kramer multiple comparisons. *n* > 22 brain hemispheres for each experimental group.

PDF-secreting LN_V_s are important not only for driving free-running locomotor rhythms in DD, but also for generating the “morning” peak of locomotor activity that anticipates lights-on in 12-h:12-h LD conditions [12,16,17,30,31]. To determine whether LN_V_ expression of membrane-tethered δ-ACTX-Hv1a interferes with the LN_V_-dependent morning activity peak, we performed a refined analysis of the average relative activity in LD of flies expressing membrane-tethered μO-MrVIA or δ-ACTX-Hv1a using *pdf-GAL4* driver. As shown in Figure 7A, control μO-MrVIA–expressing flies exhibit robust morning and evening circadian anticipatory peaks of locomotor activity. Membrane-tethered δ-ACTX-Hv1a–expressing *pdf>δ-ACTX-Hv1a* flies also exhibit robust morning and evening circadian anticipatory peaks of locomotor activity, albeit with phase-advanced morning activity (Figure 7A) compared to control *pdf>μO-MrVIA* flies. We quantified phase advance of morning activity peaks by computing an anticipation phase score for each fly, defined as the ratio of total relative activity in the 3-h period before lights-on to that after. Increase in anticipation phase scores reflects greater anticipatory activity before lights-on compared to after, thus quantifying phase advances in anticipation. As seen in Figure 7B, membrane-tethered δ-ACTX-Hv1a expression induces dose-dependent increase in morning anticipation phase score (*p* < 0.001), indicating dose-dependent phase advance of morning anticipation caused by inhibition of *para* Na^+^ channel inactivation in LN_V_ morning cells. The dose-dependent phase advance is also apparent in the shape of envelope of relative activity occurring before lights-on, being concave for *pdf>μO-MrVIA* flies and then becoming progressively more convex with increasing dose of membrane-tethered δ-ACTX-Hv1a. These effects are observed in multiple independent replicate experiments (unpublished data).

PDF has been hypothesized to be a daily phase signal transmitted from LN_V_s to other clock neurons (and potentially non-clock targets) that entrains both clock neuron cellular oscillation and locomotor rhythms [27]. To determine whether advanced phase of morning activity in flies expressing membrane-tethered δ-ACTX-Hv1a is due to altered phase of PDF secretion rhythms, we compared the daily rhythm of accumulation of PDF in the sLN_V_ dorsomedial terminals (thought to reflect daily rhythm of secretion; [18,22]) of flies expressing either δ-ACTX-Hv1a or μO-MrVIA in the LN_V_s in LD. There is a recent study demonstrating absence of detectable PDF cycling in the sLN_V_ terminals of a particular transgenic strain of flies that nonetheless still exhibit normal circadian locomotor activity [52]. However, this result is still consistent with a key role for cyclic secretion in circadian rhythms, and with cyclic PDF accumulation as a readout of cyclic secretion, as steady-state accumulation will only reflect secretion rhythms when synthesis rate and secretion rate are similar in magnitude; under conditions in which synthesis rate outpaces secretion rate, a rhythm of secretion may not result in a detectable rhythm of steady-state accumulation. Furthermore, there is functional, albeit indirect, evidence in the literature that rhythmic-activity–dependent PDF secretion is important for circadian rhythmicity [18,20,49,50].Therefore, it is reasonable to conclude that rhythmic steady-state accumulation of PDF reflects rhythmic PDF secretion.

In LD conditions, control flies expressing membrane-tethered μO-MrVIA in the LN_V_s exhibit significantly greater anti-PDF immunofluorescence in the sLN_V_ dorsomedial terminals at zeitgeber time (ZT)2 and ZT6, in comparison to the night at ZT14 and ZT18 (*p* < 0.001; Figure 7C). In contrast, PDF accumulation in the sLN_V_ dorsomedial terminals of experimental flies expressing membrane-tethered δ-ACTX-Hv1a in the LN_V_s cycles with advanced phase, peaking late night, rather than early in the morning, with peak anti-PDF immunofluorescence at around ZT22 (*p* < 0.001). This approximately 4-h phase advance of PDF cycling induced by LN_V_ expression of membrane-tethered δ-ACTX-Hv1a, in combination with the induced phase advance of morning anticipation, provides experimental support for the hypothesis that the phase of rhythmic PDF secretion determines the phase of morning anticipation. Figure S4 shows an independent replicate of this experiment, confirming δ-ACTX-Hv1a–induced phase advance of PDF accumulation.

**Figure 8:**
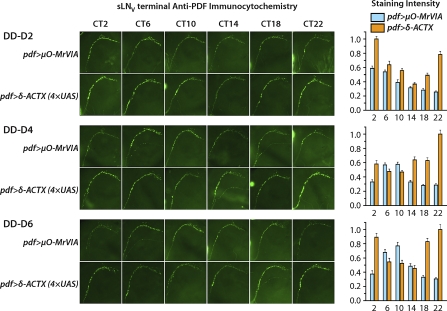
Membrane-Tethered δ-ACTX-Hv1a Expression in LN_V_ Neurons Induces Phase Advance of PDF Oscillation in sLN_V_ Dorsomedial Terminals in Constant Darkness (DD) Flies with *pdf-GAL4* and either *UAS- δ-ACTX-Hv1a* (4×UAS) or *UAS-μO-MrVIA* transgene were entrained for at least 4 d in LD and then transferred to DD. On the 2nd, 4th, or 6th d of DD, fly brains were processed for anti-PDF immunofluorescence in the sLN_V_ dorsomedial terminals. On DD-D2, control *pdf>μO-MrVIA* flies exhibit rhythmic anti-PDF immunofluorescence in the sLN_V_ dorsomedial terminals with peak accumulation at CT2 to CT6 (*p* < 0.001). In contrast, experimental *pdf>δ-ACTX-Hv1a* flies exhibit a peak at CT22 to CT2 and trough at CT14 (*p* < 0.001). On DD-D4, control *pdf>μO-MrVIA* flies exhibit a peak at CT6-CT10 (*p* < 0.001). In contrast, experimental *pdf>δ-ACTX-Hv1a* flies exhibit a peak at CT22 and trough at CT6–CT10 (*p* < 0.001). On DD-D6, PDF oscillation peaks at CT10 and trough at CT18–CT22 (*p* < 0.001) in control *pdf>μO-MrVIA* flies. In contrast, experimental *pdf>δ-ACTX-Hv1a* flies exhibit a broad peak spanning CT18, CT22, and CT2 (*p* < 0.001). Differences among different genotypes at different circadian times were compared using ANOVA with Tukey-Kramer multiple comparisons. *n* > 22 brain hemispheres for each experimental group, and error bars indicate SEM.

We also examined the effect of membrane-tethered δ-ACTX-Hv1a on accumulation of PDF in the sLN_V_ dorsomedial terminals in constant darkness. Anti-PDF immunoreactivity in the dorsomedial terminals was assayed on the 2nd, 4th, or 6th d after release from LD entraining conditions into DD. As shown in Figure 8, LN_V_ expression of membrane-tethered δ-ACTX-Hv1a induces phase advance of PDF accumulation in DD consistent with the effect in LD. On the 2nd d in DD (DD-D2), anti-PDF immunofluorescence in the sLN_V_s dorsomedial terminals of control membrane-tethered μO-MrVIA–expressing flies peaks at CT2 to CT6 (*p* < 0.001). In contrast, experimental flies expressing membrane-tethered δ-ACTX-Hv1a exhibit phase-advanced peak PDF accumulation at circadian time (CT)22 to CT2 (*p* < 0.001). On DD-D4, control *pdf>μO-MrVIA* flies exhibit peak PDF accumulation at CT6–CT10 (*p* < 0.001). Experimental *pdf>δ-ACTX-Hv1a* flies exhibit phase advance of PDF cycling with peak at CT22 (*p* < 0.001). On DD-D6, control *pdf>μO-MrVIA* flies exhibit peak PDF accumulation at CT10 (*p* < 0.001), while experimental *pdf>δ-ACTX-Hv1a* flies exhibit peak PDF accumulation around CT22 (*p* < 0.001). The gradual accumulation of a phase delay relative to the 24-h d of PDF oscillation in control *pdf>μO-MrVIA* flies over 6 d in DD is consistent with their free-running behavioral period of greater than 24 h (Figures 2 and 6). The phase advance of PDF accumulation in sLN_V_ terminals of membrane-tethered δ-ACTX-Hv1a–expressing flies in LD and DD relative to μO-MrVIA–expressing control and the correlated phase advance of morning anticipatory locomotor activity support the hypothesis that phase of LN_V_ PDF secretion rhythms determines phase of morning anticipation.

### Membrane-Tethered δ-ACTX-Hv1a Expression in LN_V_ Neurons Induces a Phase Shift between Par Domain Protein 1 (PDP1) and PDF Oscillations in sLN_V_s

The results described above show that membrane-tethered δ-ACTX-Hv1a expression in PDF-secreting LN_V_s induces a phase-advance of both sLN_V_ PDF secretion rhythms and morning anticipatory locomotor behavior in LD, and disrupts free-running locomotor rhythms in DD. In order to determine the relationship between these phenotypes and cellular transcriptional feedback oscillation, par domain protein 1 (PDP1) clock protein levels were assayed in the sLN_V_, dorsal lateral neuron (LN_D_), dorsal neuron (DN)1, and DN2 neurons of flies expressing membrane-tethered δ-ACTX-Hv1a in the LN_V_s. PDP1 is an oscillating transcription factor that has been implicated in a second interlocking circadian transcriptional feedback loop [8]. Regardless of the validity of the assertion that PDP1 does not participate in the cellular time-keeping mechanism and is solely a clock output factor [53], PDP1 is a high-fidelity phase marker for circadian transcriptional feedback oscillation and has been used as such by multiple laboratories [18,32,54,55]. Flies expressing membrane-tethered μO-MrVIA or δ-ACTX-Hv1a are entrained in LD conditions and then released into DD. Brains are fixed at different circadian times, followed by anti-PDP1 immunofluorescence. Anti-PDP1 immunofluorescence is detected in nuclei of clock neurons, and fluorescence levels are quantified by counting the number of stained nuclei in the various anatomical cell groups. To confirm the reliability of this counting method for assaying cellular transcriptional oscillation, we compared it to the established background-subtracted pixel intensity method [13,18,54]. As shown in Figure S3, these two methods reveal the identical temporal patterns of PDP1 oscillation on DD-D4 in DN1 neurons of flies expressing either membrane-tethered μO-MrVIA or δ-ACTX-Hv1a in LN_V_s. This establishes the reliability of counting PDP1-stained nuclei as a much less labor-intensive method for assessing transcriptional feedback oscillation at the cellular level.

**Figure 9:**
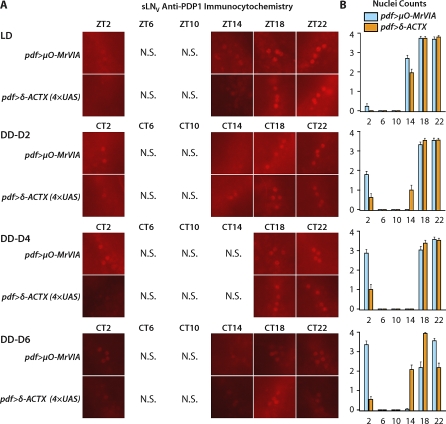
Membrane-Tethered δ-ACTX-Hv1a Expression in LN_V_ Neurons Induces No Phase Advance of PDP1 Oscillation in the sLN_V_s in LD Red anti-PDP1 immunofluorescence (A) reveals PDP1 accumulation in sLN_V_ nuclei. Bar graphs (B) show the number of neurons labeled with anti-PDP1 immunofluorescence (mean ± SEM). Representative images of sLN_V_s from flies of the indicated genotypes at the indicated time points are shown. N.S. indicates complete absence of sLN_V_ anti-PDP1 staining above background for that time point and genotype. In LD or on DD-D2, sLN_V_s of control *pdf>μO-MrVIA* flies exhibit peak PDP1 staining at ZT/CT18–ZT/CT22 (*p* < 0.001). Peak of PDP1 accumulation in sLN_V_s expressing μO-MrVIA is slightly delayed to CT18–CT22 and CT2 by DD-D4 and CT22 to CT2 by DD-D6 (*p* < 0.001). PDP1 accumulation in the sLN_V_s of *pdf>δ-ACTX-Hv1a* flies exhibits temporal pattern similar to that of control *pdf>μO-MrVIA* flies in LD and on DD-D2 and DD-D4, with peak at ZT/CT18–ZT/CT22 and trough at ZT/CT6–ZT/CT10 (*p* < 0.001). On DD-D6, peak PDP1 accumulation in the sLN_V_s of *pdf>δ-ACTX-Hv1a* flies is slightly advanced to CT18 compared with CT22–CT2 of control *pdf>μO-MrVIA* flies (*p* < 0.001). Differences among different genotypes at different circadian times were compared using ANOVA with Tukey-Kramer multiple comparisons. *n* > 22 brain hemispheres for each experimental group.

Consistent with previous studies [8,13,18,54], control *pdf>μO-MrVIA* flies expressing membrane-tethered μO-MrVIA in the LN_V_s exhibit PDP1 oscillation in sLN_V_s with peak levels late at night or subjective night and trough levels during day or subjective day (Figure 9). In LD or on DD-D2, sLN_V_s exhibit peak of PDP1 accumulation at CT18–CT22 with approximately four stained sLN_V_s detected (*p* < 0.001). PDP1 accumulation in sLN_V_s of control *pdf>μO-MrVIA* flies peaks at CT18–CT22–CT2 on DD-D4 and at CT22–CT2 on DD-D6 (*p* < 0.001). This gradually increasing slight phase delay of PDP1 accumulation in control flies free-running in DD is consistent with the free-running phase delays of PDF oscillation in the sLN_V_ dorsomedial terminals and moderately long period of free-running locomotor rhythms (Figures 2, 6, and 8, Table S1). PDP1 accumulation in the nuclei of sLN_V_s of experimental *pdf>δ-ACTX-Hv1a* flies expressing membrane-tethered δ-ACTX-Hv1a in the LN_V_s exhibits the same temporal pattern in LD as that of control flies. Thus, membrane-tethered δ-ACTX-Hv1a expression in the LN_V_s has no effect on PDP1 oscillation in sLN_V_s in LD, while inducing phase advance of PDF oscillation in their dorsomedial terminals. This phase shift between sLN_V_ PDP1 and PDF oscillations in LD induced by LN_V_ expression of membrane-tethered δ-ACTX-Hv1a was also observed in the independent replicate experiment depicted in Figure S4. The phase advance of morning anticipatory locomotor activity induced by LN_V_ expression of membrane-tethered δ-ACTX-Hv1a thus is determined by the phase of PDF rhythms and not of PDP1. In DD conditions, experimental membrane-tethered δ-ACTX-Hv1a–expressing flies exhibit similar temporal pattern of PDP1 accumulation in the nuclei of sLN_V_s as control *pdf>μO-MrVIA* flies on DD-D2 or DD-D4, with a peak at CT18–CT22 (*p* < 0.001, Figure 9). On DD-D6, PDP1 accumulation in the sLN_V_s of *pdf>δ-ACTX-Hv1a* flies peaks at CT18 (*p* < 0.001), slightly advanced compared with control *pdf>μO-MrVIA* flies. These results provide experimental support for the hypothesis that the phase of sLN_V_ PDF secretion determines the phase of morning anticipatory locomotor activity, and that PDF transmits phase information from sLN_V_s to downstream PDF-sensitive targets.

### Membrane-Tethered δ-ACTX-Hv1a Expression in LN_V_ Neurons Induces Short-Period PDP1 Oscillation in Non-LN_V_ Neurons

**Figure 10:**
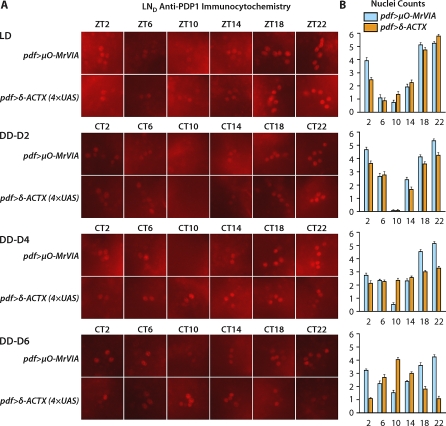
Membrane-Tethered δ-ACTX-Hv1a Expression in LN_V_ Neurons Induces Short Period of PDP1 Oscillation in LN_D_s in DD Red anti-PDP1 immunofluorescence (A) reveals PDP1 accumulation in LN_D_ nuclei. Bar graphs (B) show the number of neurons labeled with anti-PDP1 immunofluorescence (mean ± SEM). Control *pdf>μO-MrVIA* flies exhibit a similar temporal pattern of PDP1 accumulation in the LN_D_s as in the sLN_V_s, with peak centered around CT22 (*p* < 0.001). Experimental *pdf>δ-ACTX-Hv1a* flies exhibit similar temporal pattern of PDP1 oscillation to control *pdf>μO-MrVIA* flies on DD-D2 with peak at CT22 and trough at CT10. On DD-D4, LN_D_s of *pdf>δ-ACTX-Hv1a* flies exhibit damped peak of PDP1 oscillation at CT22 and trough at CT2-CT6 (*p* < 0.001). However, by DD-D6, a phase advance of PDP1 accumulation in the nuclei of LN_D_s induced by δ-ACTX-Hv1a expression manifests, with a peak at CT10 and trough at CT22-CT2 (*p* < 0.001). δ-ACTX-Hv1a expression in LN_V_s does not affect PDP1 oscillation in LN_D_ neurons in LD. Differences among different genotypes at different circadian times were compared using ANOVA with Tukey-Kramer multiple comparisons. *n* > 22 brain hemispheres for each experimental group, and error bars indicate SEM.

We used anti-PDP1 immunofluorescence to assess the effects of phase-advanced PDF cycling in δ-ACTX-Hv1a–expressing PDF-secreting LN_V_s on cellular oscillation of non-LN_V_ clock neurons that express functional PDF receptor [26]. Control *pdf>μO-MrVIA* flies exhibit a similar temporal pattern of PDP1 accumulation in the LN_D_ cell group in both LD and DD conditions, with peak levels late at night or subjective night, and trough levels during day or subjective day (Figure 10; *p* < 0.001). This constant phase of PDP1 accumulation over 6 d in DD indicates that LN_D_ cellular oscillation free-runs with approximately 24-h period in control flies. Experimental *pdf>δ-ACTX-Hv1a* flies exhibit a similar temporal pattern of LN_D_ PDP1 oscillation to control *pdf>μO-MrVIA* flies in LD and on DD-D2. However, by DD-D4, PDP1 oscillation in LN_D_s of experimental *pdf>δ-ACTX-Hv1a* flies has begun to phase shift relative to control, with blunting of peak accumulation late in subjective night (*p* < 0.001). By DD-D6, the peak of LN_D_ PDP1 accumulation in flies expressing membrane-tethered δ-ACTX-Hv1a in LN_V_s has phase advanced to CT10. This phase advance indicates that membrane-tethered δ-ACTX-Hv1a expression in the LN_V_s induces an increase in the pace of LN_D_ cellular oscillation. The approximately 12-h phase advance that accumulates after 6 d in DD is consistent with the approximately 22-h free-running period of the short-period component of the complex locomotor rhythms exhibited by many *pdf>δ-ACTX-Hv1a* flies.

**Figure 11:**
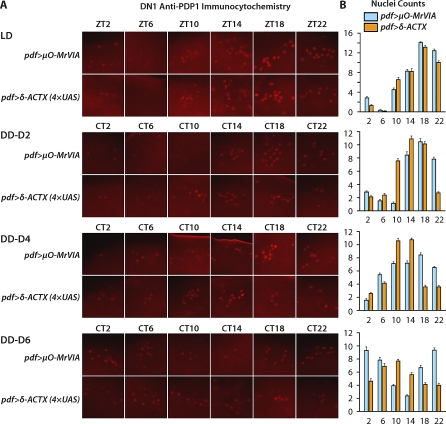
Membrane-Tethered δ-ACTX-Hv1a Expression in LN_V_ Neurons Induces Short Period of PDP1 Oscillation in DN1s in DD Red anti-PDP1 immunofluorescence (A) reveals PDP1 accumulation in DN1 nuclei. Bar graphs (B) show the number of neurons labeled with anti-PDP1 immunofluorescence (mean ± SEM). DN1s of control *pdf>μO-MrVIA* flies exhibit a peak of PDP1 accumulation at ZT18–ZT22 and trough at ZT6 (*p* < 0.001), in phase with sLN_V_s in LD conditions. In DD conditions, PDP1 oscillation in DN1s peaks at around CT18 on DD-D2 and DD-D4, and trough at CT6–CT10 on DD-D2 and CT2 on DD-D4 (*p* < 0.001). On DD-D6, PDP1 oscillation in DN1s of control flies peaks at CT22–CT2 and trough at CT14 (*p* < 0.001). In contrast, on DD-D2, experimental *pdf>δ-ACTX-Hv1a* flies exhibit PDP1 accumulation in the DN1s with peak at CT14–CT18 and trough at CT2–CT6, approximately 4 h earlier than in control flies. PDP1 accumulation in DN1s of *pdf>δ-ACTX-Hv1a* flies continuously shifts peak from CT10–CT14 by DD-D4 (*p* < 0.001) to CT6–CT10 by DD-D6 (*p*< 0.001). δ-ACTX-Hv1a expression in LN_V_s does not affect PDP1 oscillation in DN1 neurons in LD. Differences among different genotypes at different circadian times were compared using ANOVA with Tukey-Kramer multiple comparisons. *n* >22 brain hemispheres for each experimental group, and error bars indicate SEM.

In control *pdf>μO-MrVIA* flies, DN1s exhibit a peak of PDP1 accumulation in LD around ZT18 (Figure 11). In DD conditions, peak of PDP1 accumulation in DN1s of control flies broadens, but does not exhibit any phase shift. Experimental *pdf>δ-ACTX-Hv1a* flies expressing membrane-tethered δ-ACTX-Hv1a in LN_V_s exhibit a similar pattern of PDP1 oscillation as control *pdf>μO-MrVIA* flies in LD, with peak around ZT18 (*p* < 0.001). In DD, however, PDP1 oscillation of DN1s in *pdf>δ-ACTX-Hv1a* flies gradually phase advances with accumulation of an approximately 12-h delay after 6 d in DD, consistent with the approximately 22-h free-running locomotor rhythm component of complex rhythmic *pdf>δ-ACTX-Hv1a* flies.

**Figure 12:**
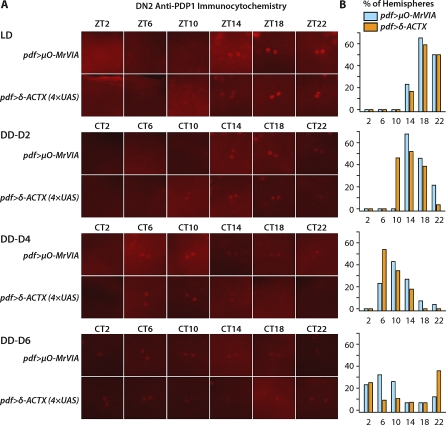
Membrane-Tethered δ-ACTX-Hv1a Expression in LN_V_ Neurons Induces Short Period of PDP1 Oscillation in DN2s in DD Red anti-PDP1 immunofluorescence (A) reveals PDP1 accumulation in DN2 nuclei. Bar graphs (B) show the percentage of brain hemispheres with an identified pair of anatomically distinguishable PDP1-positive DN2s. The DN2s of control *pdf>μO-MrVIA* flies drift out of phase with the 24-h day and the other cell groups, exhibiting peak PDP1 accumulation at CT14 on DD-D2, CT10 on DD-D4, and CT6 on DD-D6. The DN2s of experimental *pdf>δ-ACTX-Hv1a* flies exhibit a similar temporal pattern of PDP1 accumulation to control flies in LD. In DD conditions, PDP1 accumulation in DN2s of *pdf>δ-ACTX-Hv1a* flies phase advance more rapidly than control flies, with peak at CT10–CT14 on DD-D2, CT6 on DD-D4, and CT22 on DD-D6 (*p* < 0.01, χ^2^). *n* >22 brain hemispheres for each experimental group and error bars indicate SEM.

The DN2s of control *pdf>μO-MrVIA* flies exhibit peak PDP1 accumulation at ZT18–ZT22 in LD (Figure 12). The DN2s of control flies exhibit a peak of PDP1 accumulation at CT14 on DD-D2, CT10 on DD-D4, and CT6 on DD-D6. This gradual phase advance of DN2 PDP1 oscillation out of synchrony with the other cell groups after release into DD (Figures 9–12) is consistent with published observations of DN2 cellular oscillation assayed with anti-PER, anti-TIM, or anti-PDP1 immunostaining [18,54,56] and reflects inherent accelerated cellular oscillation in DN2s in DD. Experimental *pdf>δ-ACTX-Hv1a* flies also exhibit gradual phase advance of PDP1 oscillation after release into DD, peaking around CT0 on DD-D6, but this advance is greater than in control flies, which peak around CT6. Acceleration of non-LN_V_ clock neurons in free-running DD conditions has also been observed in flies in which LN_V_ membrane excitability is altered by ectopic ion channel subunit expression [18,54]. Taken together, all these results indicate that altered membrane activity of LN_V_s induced by expression of membrane-tethered δ-ACTX-Hv1a (1) phase advances sLN_V_ PDF secretion rhythms and morning anticipatory locomotor activity in LD without phase advance of sLN_V_ PDP1 oscillation and (2) accelerates free-running PDP1 oscillation of LN_D_, DN1, and DN2 PDF receptor-expressing clock neurons in DD.

## Discussion

In order to begin to dissect the contribution of particular ionic conductances to the cellular physiological function of the circadian control network, we have employed an adaptation of the tethered-toxin methodology in which peptide ion channel toxins are expressed as chimeric fusion proteins with an N-terminal secretory signal sequence and a C-terminal GPI anchor targeting signal [34]. Membrane-tethered toxins derived from blockers of vertebrate ion channels have been demonstrated to cell-autonomously block their target ion channels with expected pharmacological specificity, when transiently expressed in *Xenopus* oocytes or zebrafish muscle fibers [34]. Here, we extend this system to the use of spider toxins active against insect ion channels in transgenic Drosophila melanogaster, developing and validating four membrane-tethered spider toxins with activity against particular voltage-gated Na^+^, K^+^, and Ca^2+^ channels. Each of these four toxins causes embryonic/larval lethality when expressed pan-neuronally (Figure 1), and three of them induce behavioral phenotypes when expressed in PDF-secreting LN_V_ pacemaker neurons (Figure 2, Table S1). One of these four toxins, δ-ACTX-Hv1a, is a known inhibitor of inactivation of voltage-gated Na^+^ channels [35].

In order to elucidate mechanisms of intercellular communication in the circadian control network, we employ this novel membrane-tethered spider toxin δ-ACTX-Hv1a as a tool for cell-autonomously inhibiting *para* Na^+^ channel inactivation in vivo in PDF-secreting LN_V_ pacemaker neurons of transgenic Drosophila melanogaster. When coexpressed in *Xenopus* oocytes with *para* voltage-gated Na^+^ channel, membrane-tethered δ-ACTX-Hv1a dose-dependently inhibits rapid inactivation, with complete abolition at the highest dose (Figure 3). When expressed in PDF-secreting LN_V_ pacemaker neurons, membrane-tethered δ-ACTX-Hv1a induces a dramatic change in LN_V_ membrane excitability (Figure 5). Wild-type (WT) lLN_V_ neurons generally exhibit tonic or brief burst action potential firing patterns on a relatively stable baseline resting membrane potential (Figure 5; [49,50]). In contrast, lLN_V_ clock neurons expressing membrane-tethered δ-ACTX-Hv1a exhibit a repetitive pattern of prolonged burst firing followed by a sustained plateau potential, which is in turn followed by a massive hyperpolarization that is slowly recovered from (Figure 5). The massive hyperpolarization is likely mediated by ouabain-sensitive Na^+^/K^+^-ATPase pump current (Figure 5). The physiological effect of membrane-tethered δ-ACTX-Hv1a is explicable as the consequence of induction of a noninactivating toxin-bound subpopulation of *para* Na^+^ channels.

We have performed these electrophysiological experiments on lLN_V_s, despite the fact that it is the sLN_V_s that are thought to underlie the morning anticipatory peak of locomotor activity and dominate free-running rhythmicity in DD (for review, see [11]. This is for technical reasons relating to the very small size of the sLN_V_s and the difficulty of obtaining whole-cell electrophysiological recordings from them. It is, nonetheless, reasonable to consider the effects of membrane-tethered δ-ACTX-Hv1a on lLN_V_s as a proxy for their effects on sLN_V_s, given that the sLN_V_s are certain to possess *para* Na^+^ channels (the only known Na^+^ channel encoded in the *Drosophila* genome[57,58]) and the Na^+^/K^+^-ATPase.

The alteration of LN_V_ membrane activity induced by expression of membrane-tethered δ-ACTX-Hv1a has profound effects on circadian rhythms at the cellular and organismal level. In LD conditions, LN_V_ expression of membrane-tethered δ-ACTX-Hv1a induces phase advance of both sLN_V_ terminal PDF cycling and the morning anticipatory peak of locomotor activity (Figure 7, Figure S4), but does not alter phase of sLN_V_ transcriptional feedback oscillation (Figure 9, Figure S4). In DD, LN_V_ expression of membrane-tethered δ-ACTX-Hv1a induces arrhythmic and complex behavioral rhythms (Figures 2 and 6), phase advance of sLN_V_ PDF rhythms (Figures 7 and 8), and acceleration of cellular oscillation in non-LN_V_ clock cells (Figures 10–12). It is interesting that the same manipulation induces a phase advance of morning activity in LD, while inducing complex and arrhythmic phenotypes in DD. The likely reason for this is that in LD, phase of transcriptional oscillation is dominated by cell-autonomous CRYPTOCHROME-dependent entrainment to the LD cycle [59–61]—thus synchronizing transcriptional rhythms in all clock neurons—whereas in DD, this strong synchronous entraining cue is absent.

These results lead to two important novel conclusions about the relationship between transcriptional feedback oscillation, membrane activity, rhythmic PDF secretion, and behavioral rhythms. First, our results support the conclusion that transcriptional feedback oscillation sets phase of rhythmic PDF secretion via modulation of membrane activity, as manipulation of LN_V_ membrane activity by inhibition of Na^+^ channel inactivation alters the phase relationship between sLN_V_ transcriptional oscillation and sLN_V_ PDF rhythms. The specific mechanism by which inhibition of *para* inactivation alters the phase relationship between transcriptional and peptide output rhythms is an important issue for future studies, as it is likely to shed light on the mechanisms by which transcriptional oscillation influences membrane activity [49]. Interestingly, LN_V_ expression of membrane-tethered δ-ACTX-Hv1a also induces an increase in the amplitude of sLN_V_ PDF rhythms (Figures 7 and 8); the reason for an increase in amplitude likely relates to this mechanism of transcription-activity coupling.

Second, our results provide experimental support for the hypothesis that sLN_V_ M cells communicate phase information to non-LN_V_ clock cells, and set the phase of behavioral rhythms, using rhythmic PDF secretion as the phase signal. Previous studies have shown that injected PDF can alter phase of cockroach locomotor rhythms [29] and that *Drosophila* PDF-secreting LN_V_s reset the phase daily both of some non-LN_V_ clock neurons and of locomotor rhythms [27]. Here, we show that phase of sLN_V_ PDF cycling determines phase of morning anticipation and non-LN_V_ clock neuron cellular oscillation, thus establishing rhythmic PDF secretion by sLN_V_s as an entraining phase signal in the circadian control network.

The behavioral and cellular effects of δ-ACTX-Hv1a in DD raise the interesting question as to the precise cellular basis for the complex behavioral rhythms also induced by other manipulations of PDF signaling in the circadian control circuit [18,20,62]. Although it does not appear that any of the cell groups we have assayed via anti-PDP1 immunostaining exhibit detectable long-period cellular rhythms that would be consistent with the approximately 25-h long-period component of the complex behavioral rhythms (Table S1), it is possible that some subtypes of LN_D_s and/or DN1s are differentially affected by PDF signals, so as to induce short-period or long-period oscillations in few, or even single, neurons that are undetectable in the context of the anatomical group as a whole. As a corollary, it is possible that PDF has period-lengthening effects on some clock neurons and period shortening effects on others. Differences in the cellular consequences of manipulations that result in similar behavioral complex rhythms may thus be explained by the possibility that there are multiple free-running configurations of the circadian control circuit that result in similar behavioral complex rhythms—such that in different configurations, it is different particular subgroups of clock neurons that control long or short period locomotor rhythms.

These studies have a number of implications for further inquiry. The specific mechanism by which inhibition of *para* inactivation alters the phase relationship between transcriptional and peptide output rhythms is an important issue for future studies. In relation to the role of PDF as a phase signal from sLN_V_ M cells to non-LN_V_ clock neurons and, possibly, other downstream targets, future studies are required to determine the identity of the particular PDF receptor-expressing cells that transduce the phase of sLN_V_ PDF secretion into phase of morning anticipation. In relation to the tethered-toxin technology, our studies now establish the feasibility of this approach to cellular dissection of ion channel function in genetically targeted cellular subsets in the nervous system of a transgenic animal. The four membrane-tethered spider toxins we have developed and validated can now be applied to analysis of ion channel function in other circuits in the *Drosophila* nervous system. Future studies are also required to determine the cellular and biophysical basis of interference with circadian function by LN_V_ expression of membrane-tethered ω-ACTX-Hv1c and κ-ACTX-Hv1c.

## Materials and Methods

### Fly strains.

*pdf-GAL4* and *pdf-gal4;UAS-DsRedII* fly lines are as described previously [17,54,63]. *pdf-GAL4* was recombined with *tubGAL80^ts^* (from Bloomington *Drosophila* Stock Center) on the second chromosome. *UAS-toxin* fly strains are generated via standard P element transgenesis techniques [64]. Membrane-tethered toxin transgenes were chemically synthesized with optimal *Drosophila* codon usage and an optimal *Drosophila* Kozak translation initiation sequence [AAA]. Encoded chimeric membrane-tethered toxin sequence is based on the long-linker version of Ibañez-Tallon et al. [34], except Myc epitope tag is substituted for FLAG. *pdf-GAL4* or *pdf-GAL4,tubGAL80^ts^* virgins were crossed to *UAS* males to generate male progeny for behavioral analysis or immunostaining. *pdf-GAL4;UAS-DsRedII* virgins were crossed with *UAS* males to generate progeny for electrophysiological recordings. Multiple independent chromosomal insertions of the *UAS-toxin* transgene were recombined using classical genetic methods to generate second and third chromosomes bearing two independent insertions each for dose-dependent experiments.

### Circadian behavioral analysis.

Locomotor activity of individual adult male flies (1–4 d posteclosion) was measured at 25 °C using the TriKinetics infrared beam-crossing system. The flies were first monitored for 5∼6 d in 12-h:12-h LD conditions, and free-running locomotor activity was then monitored in constant darkness (DD) for two further weeks. Total infrared beam crosses in 10-min bins were recorded and circadian rhythms were analyzed in 20-min blocks by Lomb-Scargle periodogram using Actimetrics Clocklab software [65]. Significant circadian rhythmicity was defined as presence of a peak in periodogram power that exceeds the *p* = 0.05 significance line, and the percentages of arrhythmic flies were compared using chi-square analyses. Rhythm strength is defined by the height of the relevant periodogram peak, expressed in arbitrary units. Average numbers of activity events per half-hour bin per fly were also calculated, and histograms for relative activities were generated for LD behavior of the flies among 5 d. In addition, the morning anticipation phase score in LD was computed as relative activity in 3-h period before versus after lights-on transition for individual flies, and these peak phases among different genotypes were statistically analyzed by the ANOVA with Tukey-Kramer multiple comparisons.

### Two-electrode voltage clamp recording of *Xenopus* oocytes.

δ-ACTX-Hv1a and PLTXII were subcloned into *pCS2+* plasmid for *Xenopus* oocyte expression. The gene constructs for cloning *Drosophila* voltage-gated Na^+^ channel α subunit (para) and its auxiliary β subunit (tipE), *pGH19-13-5 para* [47,48] and *pGH tipE*, are from M. Williamson (Rothamsted Research, Harpenden, United Kingdom). *pBSc-ShH37* for *Drosophila* voltage-gated K^+^ channel, Shaker, is from L. Salkoff (Washington University, St. Louis, Missouri). Xenopus laevis oocytes were prepared, injected, and recorded using standard methods in ND96 solution [66]. Oocytes were injected with less than 50 nl in vitro transcribed cRNAs encoding para/tipE and either δ-ACTX-Hv1a or PLTXII. The full-strength (1:1) of para, tipE, δ-ACTX-Hv1a, and PLTXII cRNAs are 1.66 ng/nl, 1.32 ng/nl, 2.24 ng/nl, and 1.95 ng/nl, respectively. δ-ACTX-Hv1a cRNA was diluted into the various dilutions (1:10, 1:100, 1:1,000, et al.). Then para, tipE, and either δ-ACTX-Hv1a or PLTXII cRNAs were mixed at 1:1:1 ratio of volume and injected into the oocytes. Two days after para/tipE cRNA injection, inward Na^+^ current evoked by 40-ms or 100-ms depolarizing steps from −70 mV to 40 mV with 10-mV increments at the holding potential of −90 mV was recorded using two-electrode voltage clamp. The full strength of 2.89 ng/nl Shaker cRNA was diluted 10-fold and mixed with equal volume of H_2_O or 1:1 δ-ACTX-Hv1a cRNA. Three days after Shaker cRNA injection, outward K^+^ current evoked by 400-ms depolarizing steps from the holding potential of −90 mV was recorded.

### Clock neuron electrophysiology.

Whole-cell recordings on lLN_V_ of fly brain explants are performed as described [49,54]. Briefly, the brains of flies 3–7 d posteclosion are dissected in external recording solution, which consists of (in mM): 101 NaCl, 3 KCl, 1 CaCl_2_, 4 MgCl_2_, 1.25 NaH_2_PO_4_, 5 glucose, 20.7 NaHCO_3_ (pH 7.2) with osmolarity of 250 mmol/kg. The brain is placed ventral side up and secured in a recording chamber with a mammalian brain-slice “harp” holder and is continuously perfused with external solution bubbled with 95% O_2_/5% CO_2_ at room temperature. LN_v_s are visualized by DsRed fluorescence, and the immediate area surrounding the lLN_V_s is enzymatically digested with focal application of protease XIV (2 mg/ml, Sigma). Whole-cell recordings are performed using borosilicate standard-wall capillary glass pipettes (Sutter Instrument Company), and data are acquired with Axopatch 200B amplifier, Digidata 1200 A/D hardware, and pClamp 8.0 software (Axon Instruments). Recording pipettes are filled with internal solution consisting of (in millimolar): 102 potassium gluconate, 17 NaCl, 0.085 CaCl_2,_ 4 Mg-ATP, 0.5 Na-GTP, 0.94 EGTA, and 8.5 HEPES (pH 7.2) and osmolarity of 235 mmol/kg. Gigaohm seals are achieved before recording in cell-attached configuration in voltage-clamp mode, followed by break-in to whole-cell configuration. Then current-clamp is employed to monitor RMP and AP firing rate.

### Brain immunocytochemistry.

Adult fly brains were dissected and processed for anti-PDP1 (1:2,000, rabbit) and anti-PDF (1:50, mouse) immunocytochemistry as described previously [18,54]. PDP1 and PDF were visualized using a Cy3-conjugated anti-rabbit secondary antibody (1:200) and a Cy2-conjugated anti-mouse secondary antibody (1:200). Because the DN2 subgroup of clock neurons is frequently indistinguishable anatomically from the nearby DN1 clock neurons, DN2 staining was scored for each brain hemisphere only as the presence or absence of a distinct pair of neurons ventral to the DN1s, and analyzed by comparing the percentage of brain hemispheres exhibiting such a distinct pair. Anti-PDP1 and anti-PDF immunofluorescent images were collected using a charge-coupled device (CCD) camera mounted on a Zeiss Axiophot epifluorescent photomicroscope and used for analysis. In our previous studies, the average background pixel value for each image was subtracted from the threshold-selected pixels of that image to yield the final threshold-selected background-subtracted images. The integrated pixel values of the threshold-selected background-subtracted images were normalized within each cell group and genotype in LD cycle or DD to the time point with the greatest average integrated pixel value. In this study, a simpler and much less labor-intensive method was used to analyze the PDP1 staining pattern, wherein recognizable nuclei exhibiting anti-PDP1 antibody are counted. We have validated that this counting method reveals identical temporal pattern of PDP1 nucleus accumulation as the previous staining-intensity method (Figure S3). The statistical analysis of anti-PDP1 staining was performed using ANOVA with Tukey-Kramer multiple comparisons.

For dose-dependent expression of toxin δ-ACTX-Hv1a in the LN_V_s and LN_V_ terminals, adult fly brains were processed with anti-Myc (1:200, mouse) and followed by Cy3-conjugated anti-mouse secondary antibody (1:200). For colocalization of PDF and δ-ACTX-Hv1a expression in the LN_V_s and LN_V_ terminals, adult fly brains were processed with anti-Myc and anti-PDF (1:2,000, rabbit) accompanied with Cy3-conjugated anti-mouse (1:200) and Cy2-conjugated anti-rabbit (1:200) secondary antibodies. For expression of other toxins, the primary antibodies are the same, and the secondary antibodies are Cy2 (1:200) and Texas-red (1:300)-conjugated antibodies, respectively. Anti-myc (clone 9E10) is from Upstate Cell Signaling Solutions. Cy2-, Cy3-, and Texas-red–conjugated secondary antibodies are from Jackson ImmunoResearch Laboratories.

## Supporting Information

Figure S1 Membrane-Tethered ω-ACTX-Hv1c Localization in sLN_V_ Dorsomedial Terminals *pdf -GAL4* driver flies were crossed to flies with *UAS* encoding Myc-tagged membrane-tethered *ω-ACTX-Hv1c* transgene. Adult brains of *pdf>ω-ACTX-Hv1c* flies or *UAS*-*ω-ACTX-Hv1c* flies were processed for immunofluorescence with anti-Myc and anti-PDF antibodies. Green anti-Myc staining in the brains of ω-ACTX-Hv1c–expressing flies colocalized with red anti-PDF neuropeptide in the LN_V_ cell bodies and throughout their terminals. Control *UAS-ω-ACTX-Hv1c* fly brains exhibit no LN_V_ anti-Myc staining. High resolution confocal images of the dorsomedial terminals of sLN_V_s for the inset area show that green anti-Myc fluorescence signal surrounds “puncta” of red anti-PDF signal. This is consistent with targeting of ω-ACTX-Hv1c to the plasma membrane that encloses regions of concentration of PDF-containing dense-core vesicles.
(5.76 MB PDF)

Figure S2 Disrupted Circadian Behavior Caused by Membrane-Tethered δ-ACTX-Hv1a Expression Is Not Due to Developmental Effects *pdf-GAL4,tub-GAL80^ts^* driver flies were crossed to *UAS-toxin* transgenic flies to generate progeny. Membrane-tethered δ-ACTX-Hv1a or μO-MrVIA expression was induced by transferring progeny from 18 °C to 30 °C after eclosion, the temperature that inactivates *tub-GAL80^ts^* and allows for *GAL4*-induced *UAS-toxin* expression.
(A) The averaged actograms of all flies of the indicated genotypes are shown. Flies expressing μO-MrVIA exhibit a single rhythm of free-running locomotor activity. In contrast, flies expressing δ-ACTX-Hv1a display free-running complex rhythmicity.
(B) Percentages of flies exhibiting arrhythmic (red), complex rhythmic (yellow), and single rhythmic (blue) locomotor activity assayed over the first 14 d in DD at 30 °C. *n*, number of flies tested. The difference in proportion of behavioral phenotypes between each of control flies, either *pdf,tub>μO-MrVIA* or parental *UAS-toxin* strain or *pdf,tub* driver flies, and experimental *pdf,tub>δ-ACTX-Hv1a* flies are all statistically significant; for each of the χ^2^ values, *p* < 0.001.
(485 KB PDF)

Figure S3 Cell-Counting Method Reveals Identical Time Course of Rhythmic PDP1 Accumulation as Background-Subtracted Pixel Intensity Method Bar graphs show normalized integrated anti-PDP1-staining intensities (mean ± SEM) in DN1s (A) and anti-PDP1–stained DN1 nuclei counts (B) of the indicated genotypes at the indicated time points on DD-D4. Control *pdf>μO-MrVIA* flies exhibit PDP1 oscillation in DN1s with peak at CT18 and trough at CT2 (*p* < 0.001). PDP1 accumulation in DN1s of experimental *pdf>δ-ACTX-Hv1a* flies exhibit peak at CT10–CT14 and trough at CT2 (*p* < 0.001).
(62 KB PDF)

Figure S4 Repeat Experiment Confirms Phase Shift between PDF and PDP1 Rhythms Induced by Membrane-Tethered δ-ACTX-Hv1a Expression in PDF-Secreting LN_V_ Neurons in LD Conditions Bar graphs show anti-PDP1-staining sLN_V_ nuclei counts and normalized integrated anti-PDF–staining intensities in sLN_V_ terminals (mean ± SEM) of the indicated genotypes at the indicated time points in LD. This repeat experiment demonstrates the same result as the experiment shown in Figures 7 and 9. LN_V_ expression of membrane-tethered δ-ACTX-Hv1a has no effect on phase and amplitude of PDP1 oscillation, but induces phase shift of PDF accumulation in the sLN_V_ dorsomedial terminals, with peak at ZT18–ZT22–ZT2 compared with peak at ZT2 in control flies. *n* > 14 brain hemispheres for each genotype–time point group.
(60 KB PDF)

Table S1 Period and Power of Rhythmicity in Different Genotypes of Flies (62 KB DOC)

## Abbreviations

AP: action potential
cRNA: complementary RNA
CT: circadian time
DD: constant darkness
DN: dorsal neuron
E cell: evening cell
GPI: glycosylphosphatidylinositol
LD: light:dark
lLNV: large ventral lateral neuron
LND: dorsal lateral neuron
LNV: ventral lateral neuron
M cell: morning cell
RMP: resting membrane potential
PDF: pigment dispersing factor
PDP1: par domain protein 1
SEM: standard error of the mean
sLNV: small ventral lateral neuron
WT: wild-type
ZT: zeitgeber time

## References

[pbio-0060273-b001] Sehgal A, Ousley A, Yang Z, Chen Y, Schotland P (1999). What makes the circadian clock tick: genes that keep time. Recent Prog Horm Res.

[pbio-0060273-b002] Young MW, Kay SA (2001). Time zones: a comparative genetics of circadian clocks. Nat Rev Genet.

[pbio-0060273-b003] Sehgal A, Price JL, Man B, Young MW (1994). Loss of circadian behavioral rhythms and per RNA oscillations in the Drosophila mutant timeless. Science.

[pbio-0060273-b004] Sehgal A, Rothenfluh-Hilfiker A, Hunter-Ensor M, Chen Y, Myers MP (1995). Rhythmic expression of timeless: a basis for promoting circadian cycles in period gene autoregulation. Science.

[pbio-0060273-b005] Gekakis N, Saez L, Delahaye-Brown AM, Myers MP, Sehgal A (1995). Isolation of timeless by PER protein interaction: defective interaction between timeless protein and long-period mutant PERL. Science.

[pbio-0060273-b006] Myers MP, Wager-Smith K, Wesley CS, Young MW, Sehgal A (1995). Positional cloning and sequence analysis of the Drosophila clock gene, timeless. Science.

[pbio-0060273-b007] Blau J, Young MW (1999). Cycling vrille expression is required for a functional Drosophila clock. Cell.

[pbio-0060273-b008] Cyran SA, Buchsbaum AM, Reddy KL, Lin MC, Glossop NR (2003). vrille, Pdp1, and dClock form a second feedback loop in the Drosophila circadian clock. Cell.

[pbio-0060273-b009] Reppert SM, Weaver DR (2000). Comparing clockworks: mouse versus fly. J Biol Rhythms.

[pbio-0060273-b010] Williams JA, Sehgal A (2001). Molecular components of the circadian system in Drosophila. Annu Rev Physiol.

[pbio-0060273-b011] Nitabach MN, Taghert PH (2008). Organization of the Drosophila circadian control circuit. Curr Biol.

[pbio-0060273-b012] Nitabach MN, Blau J, Holmes TC (2002). Electrical silencing of Drosophila pacemaker neurons stops the free-running circadian clock. Cell.

[pbio-0060273-b013] Harrisingh MC, Wu Y, Lnenicka GA, Nitabach MN (2007). Intracellular Ca2+ regulates free-running circadian clock oscillation in vivo. J Neurosci.

[pbio-0060273-b014] Blanchardon E, Grima B, Klarsfeld A, Chelot E, Hardin PE (2001). Defining the role of Drosophila lateral neurons in the control of circadian rhythms in motor activity and eclosion by targeted genetic ablation and PERIOD protein overexpression. Eur J Neurosci.

[pbio-0060273-b015] Kaneko M, Park JH, Cheng Y, Hardin PE, Hall JC (2000). Disruption of synaptic transmission or clock-gene-product oscillations in circadian pacemaker cells of Drosophila cause abnormal behavioral rhythms. J Neurobiol.

[pbio-0060273-b016] Helfrich-Forster C (1998). Robust circadian rhythmicity of Drosophila melanogaster requires the presence of lateral neurons: a brain-behavioral study of disconnected mutants. J Comp Physiol [A].

[pbio-0060273-b017] Renn SC, Park JH, Rosbash M, Hall JC, Taghert PH (1999). A pdf neuropeptide gene mutation and ablation of PDF neurons each cause severe abnormalities of behavioral circadian rhythms in Drosophila. Cell.

[pbio-0060273-b018] Nitabach MN, Wu Y, Sheeba V, Lemon WC, Strumbos J (2006). Electrical hyperexcitation of lateral ventral pacemaker neurons desynchronizes downstream circadian oscillators in the fly circadian circuit and induces multiple behavioral periods. J Neurosci.

[pbio-0060273-b019] Peng Y, Stoleru D, Levine JD, Hall JC, Rosbash M (2003). Drosophila free-running rhythms require intercellular communication. PLoS Biol.

[pbio-0060273-b020] Helfrich-Forster C, Tauber M, Park JH, Muhlig-Versen M, Schneuwly S (2000). Ectopic expression of the neuropeptide pigment-dispersing factor alters behavioral rhythms in Drosophila melanogaster. J Neurosci.

[pbio-0060273-b021] Lin Y, Stormo GD, Taghert PH (2004). The neuropeptide pigment-dispersing factor coordinates pacemaker interactions in the Drosophila circadian system. J Neurosci.

[pbio-0060273-b022] Park JH, Helfrich-Forster C, Lee G, Liu L, Rosbash M (2000). Differential regulation of circadian pacemaker output by separate clock genes in Drosophila. Proc Natl Acad Sci U S A.

[pbio-0060273-b023] Hyun S, Lee Y, Hong ST, Bang S, Paik D (2005). Drosophila GPCR Han is a receptor for the circadian clock neuropeptide PDF. Neuron.

[pbio-0060273-b024] Lear BC, Merrill CE, Lin JM, Schroeder A, Zhang L (2005). A G protein-coupled receptor, Groom-of-PDF, is required for PDF neuron action in circadian behavior. Neuron.

[pbio-0060273-b025] Mertens I, Vandingenen A, Johnson EC, Shafer OT, Li W (2005). PDF receptor signaling in Drosophila contributes to both circadian and geotactic behaviors. Neuron.

[pbio-0060273-b026] Shafer OT, Kim DJ, Dunbar-Yaffe R, Nikolaev VO, Lohse MJ (2008). Widespread receptivity to neuropeptide PDF throughout the neuronal circadian clock network of Drosophila revealed by real-time cyclic AMP imaging. Neuron.

[pbio-0060273-b027] Stoleru D, Peng Y, Nawathean P, Rosbash M (2005). A resetting signal between Drosophila pacemakers synchronizes morning and evening activity. Nature.

[pbio-0060273-b028] Stoleru D, Nawathean P, Fernandez Mde L, Menet JS, Ceriani MF (2007). The Drosophila circadian network is a seasonal timer. Cell.

[pbio-0060273-b029] Petri B, Stengl M (1997). Pigment-dispersing hormone shifts the phase of the circadian pacemaker of the cockroach Leucophaea maderae. J Neurosci.

[pbio-0060273-b030] Grima B, Chelot E, Xia R, Rouyer F (2004). Morning and evening peaks of activity rely on different clock neurons of the Drosophila brain. Nature.

[pbio-0060273-b031] Stoleru D, Peng Y, Agosto J, Rosbash M (2004). Coupled oscillators control morning and evening locomotor behaviour of Drosophila. Nature.

[pbio-0060273-b032] Murad A, Emery-Le M, Emery P (2007). A subset of dorsal neurons modulates circadian behavior and light responses in Drosophila. Neuron.

[pbio-0060273-b033] Picot M, Cusumano P, Klarsfeld A, Ueda R, Rouyer F (2007). Light activates output from evening neurons and inhibits output from morning neurons in the Drosophila circadian clock. PLoS Biol.

[pbio-0060273-b034] Ibañez-Tallon I, Wen H, Miwa JM, Xing J, Tekinay AB (2004). Tethering naturally occurring Peptide toxins for cell-autonomous modulation of ion channels and receptors in vivo. Neuron.

[pbio-0060273-b035] Grolleau F, Stankiewicz M, Birinyi-Strachan L, Wang XH, Nicholson GM (2001). Electrophysiological analysis of the neurotoxic action of a funnel-web spider toxin, delta-atracotoxin-HV1a, on insect voltage-gated Na+ channels. J Exp Biol.

[pbio-0060273-b036] Maggio F, King GF (2002). Scanning mutagenesis of a Janus-faced atracotoxin reveals a bipartite surface patch that is essential for neurotoxic function. J Biol Chem.

[pbio-0060273-b037] Wang X, Connor M, Smith R, Maciejewski MW, Howden ME (2000). Discovery and characterization of a family of insecticidal neurotoxins with a rare vicinal disulfide bridge. Nat Struct Biol.

[pbio-0060273-b038] Wang X, Smith R, Fletcher JI, Wilson H, Wood CJ (1999). Structure-function studies of omega-atracotoxin, a potent antagonist of insect voltage-gated calcium channels. Eur J Biochem.

[pbio-0060273-b039] Chong Y, Hayes JL, Sollod B, Wen S, Wilson DT (2007). The omega-atracotoxins: selective blockers of insect M-LVA and HVA calcium channels. Biochem Pharmacol.

[pbio-0060273-b040] Branton WD, Kolton L, Jan YN, Jan LY (1987). Neurotoxins from Plectreurys spider venom are potent presynaptic blockers in Drosophila. J Neurosci.

[pbio-0060273-b041] Leung HT, Branton WD, Phillips HS, Jan L, Byerly L (1989). Spider toxins selectively block calcium currents in Drosophila. Neuron.

[pbio-0060273-b042] Terlau H, Stocker M, Shon KJ, McIntosh JM, Olivera BM (1996). MicroO-conotoxin MrVIA inhibits mammalian sodium channels, but not through site I. J Neurophysiol.

[pbio-0060273-b043] McGuire SE, Le PT, Osborn AJ, Matsumoto K, Davis RL (2003). Spatiotemporal rescue of memory dysfunction in Drosophila. Science.

[pbio-0060273-b044] McGuire SE, Mao Z, Davis RL (2004). Spatiotemporal gene expression targeting with the TARGET and gene-switch systems in Drosophila. Sci STKE.

[pbio-0060273-b045] Ramaswami M, Tanouye MA (1989). Two sodium-channel genes in Drosophila: implications for channel diversity. Proc Natl Acad Sci U S A.

[pbio-0060273-b046] Hong CS, Ganetzky B (1994). Spatial and temporal expression patterns of two sodium channel genes in Drosophila. J Neurosci.

[pbio-0060273-b047] Feng G, Deak P, Chopra M, Hall LM (1995). Cloning and functional analysis of TipE, a novel membrane protein that enhances Drosophila para sodium channel function. Cell.

[pbio-0060273-b048] Warmke JW, Reenan RA, Wang P, Qian S, Arena JP (1997). Functional expression of Drosophila para sodium channels. Modulation by the membrane protein TipE and toxin pharmacology. J Gen Physiol.

[pbio-0060273-b049] Cao G, Nitabach MN (2008). Circadian control of membrane excitability in Drosophila melanogaster lateral ventral clock neurons. J Neurosci.

[pbio-0060273-b050] Sheeba V, Gu H, Sharma VK, O'Dowd DK, Holmes TC (2008). Circadian- and light-dependent regulation of resting membrane potential and spontaneous action potential firing of Drosophila circadian pacemaker neurons. J Neurophysiol.

[pbio-0060273-b051] Lebovitz RM, Takeyasu K, Fambrough DM (1989). Molecular characterization and expression of the (Na+ + K+)-ATPase alpha-subunit in Drosophila melanogaster. EMBO J.

[pbio-0060273-b052] Kula E, Levitan ES, Pyza E, Rosbash M (2006). PDF cycling in the dorsal protocerebrum of the Drosophila brain is not necessary for circadian clock function. J Biol Rhythms.

[pbio-0060273-b053] Benito J, Zheng H, Hardin PE (2007). PDP1epsilon functions downstream of the circadian oscillator to mediate behavioral rhythms. J Neurosci.

[pbio-0060273-b054] Wu Y, Cao G, Nitabach MN (2008). Electrical silencing of PDF neurons advances the phase of non-PDF clock neurons in Drosophila. J Biol Rhythms.

[pbio-0060273-b055] Lim C, Chung BY, Pitman JL, McGill JJ, Pradhan S (2007). Clockwork orange encodes a transcriptional repressor important for circadian-clock amplitude in Drosophila. Curr Biol.

[pbio-0060273-b056] Veleri S, Brandes C, Helfrich-Forster C, Hall JC, Stanewsky R (2003). A self-sustaining, light-entrainable circadian oscillator in the Drosophila brain. Curr Biol.

[pbio-0060273-b057] Littleton JT, Ganetzky B (2000). Ion channels and synaptic organization: analysis of the Drosophila genome. Neuron.

[pbio-0060273-b058] Zhou W, Chung I, Liu Z, Goldin AL, Dong K (2004). A voltage-gated calcium-selective channel encoded by a sodium channel-like gene. Neuron.

[pbio-0060273-b059] Stanewsky R, Kaneko M, Emery P, Beretta B, Wager-Smith K (1998). The cryb mutation identifies cryptochrome as a circadian photoreceptor in Drosophila. Cell.

[pbio-0060273-b060] Emery P, Stanewsky R, Helfrich-Forster C, Emery-Le M, Hall JC (2000). Drosophila CRY is a deep brain circadian photoreceptor. Neuron.

[pbio-0060273-b061] Emery P, So WV, Kaneko M, Hall JC, Rosbash M (1998). CRY, a Drosophila clock and light-regulated cryptochrome, is a major contributor to circadian rhythm resetting and photosensitivity. Cell.

[pbio-0060273-b062] Sheeba V, Sharma VK, Gu H, Chou YT, O'Dowd DK (2008). Pigment dispersing factor-dependent and -independent circadian locomotor behavioral rhythms. J Neurosci.

[pbio-0060273-b063] Brand AH, Perrimon N (1993). Targeted gene expression as a means of altering cell fates and generating dominant phenotypes. Development.

[pbio-0060273-b064] Rubin GM, Spradling AC (1982). Genetic transformation of Drosophila with transposable element vectors. Science.

[pbio-0060273-b065] Van Dongen HP, Olofsen E, VanHartevelt JH, Kruyt EW (1999). A procedure of multiple period searching in unequally spaced time-series with the Lomb-Scargle method. Biol Rhythm Res.

[pbio-0060273-b066] Goldin AL (1992). Maintenance of Xenopus laevis and oocyte injection. Methods Enzymol.

