# Supplementary material for: Phase Coupling of a Circadian Neuropeptide With Rest/Activity Rhythms Detected Using a Membrane-Tethered Spider Toxin
Source: PLoS Biol. 2008 Nov 4;6(11):e273. doi: 10.1371/journal.pbio.0060273 (PMC2577701; doi:10.1371/journal.pbio.0060273)
Supplement: Supplementary file 1 [file pbio.0060273.sg001.pdf]

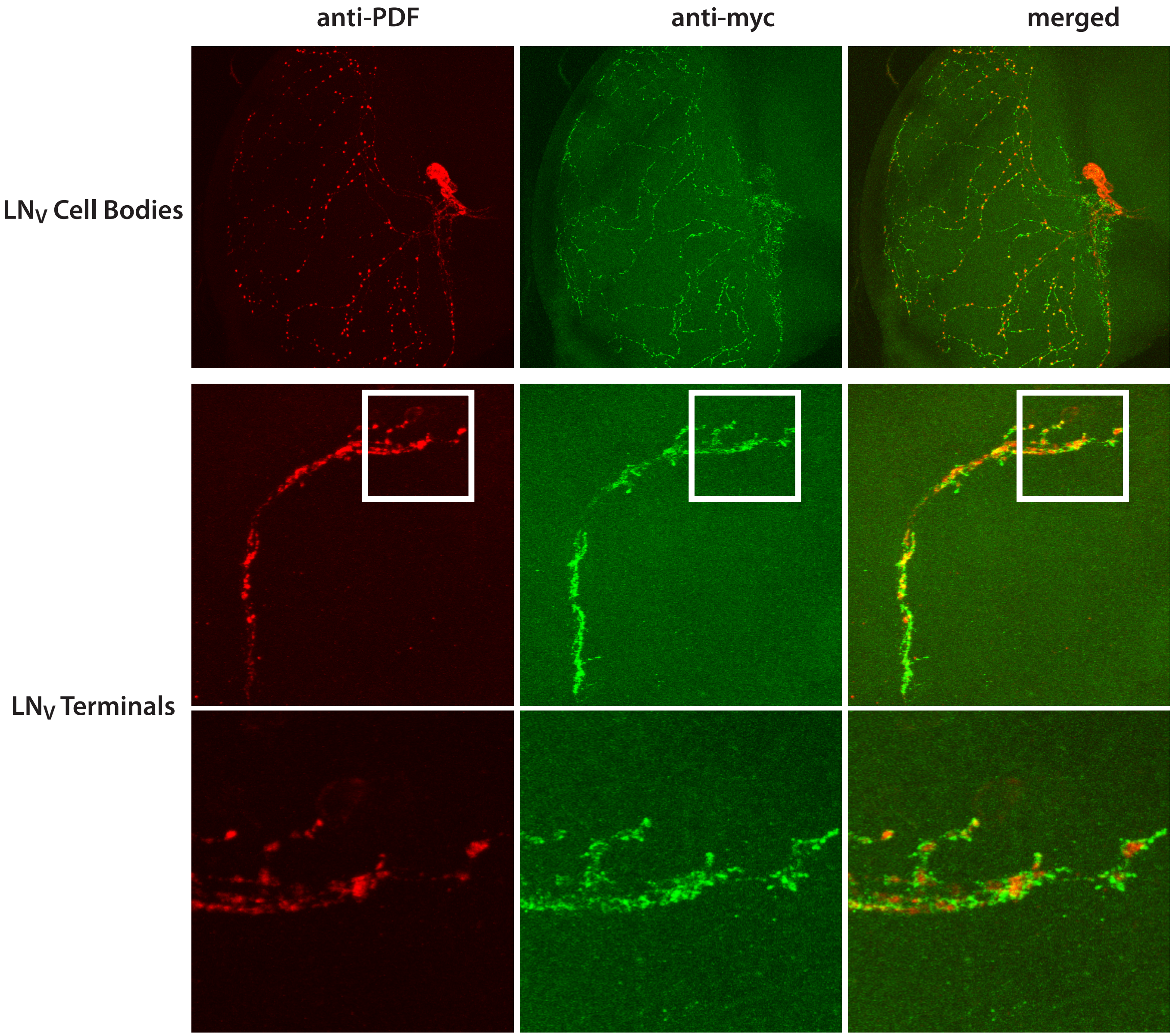

anti-PDF
anti-myc
merged
LN$_V$ Cell Bodies
LN$_V$ Terminals
