# Supplementary material for: Phase Coupling of a Circadian Neuropeptide With Rest/Activity Rhythms Detected Using a Membrane-Tethered Spider Toxin
Source: PLoS Biol. 2008 Nov 4;6(11):e273. doi: 10.1371/journal.pbio.0060273 (PMC2577701; doi:10.1371/journal.pbio.0060273)
Supplement: Supplementary file 2 [file pbio.0060273.sg002.pdf]

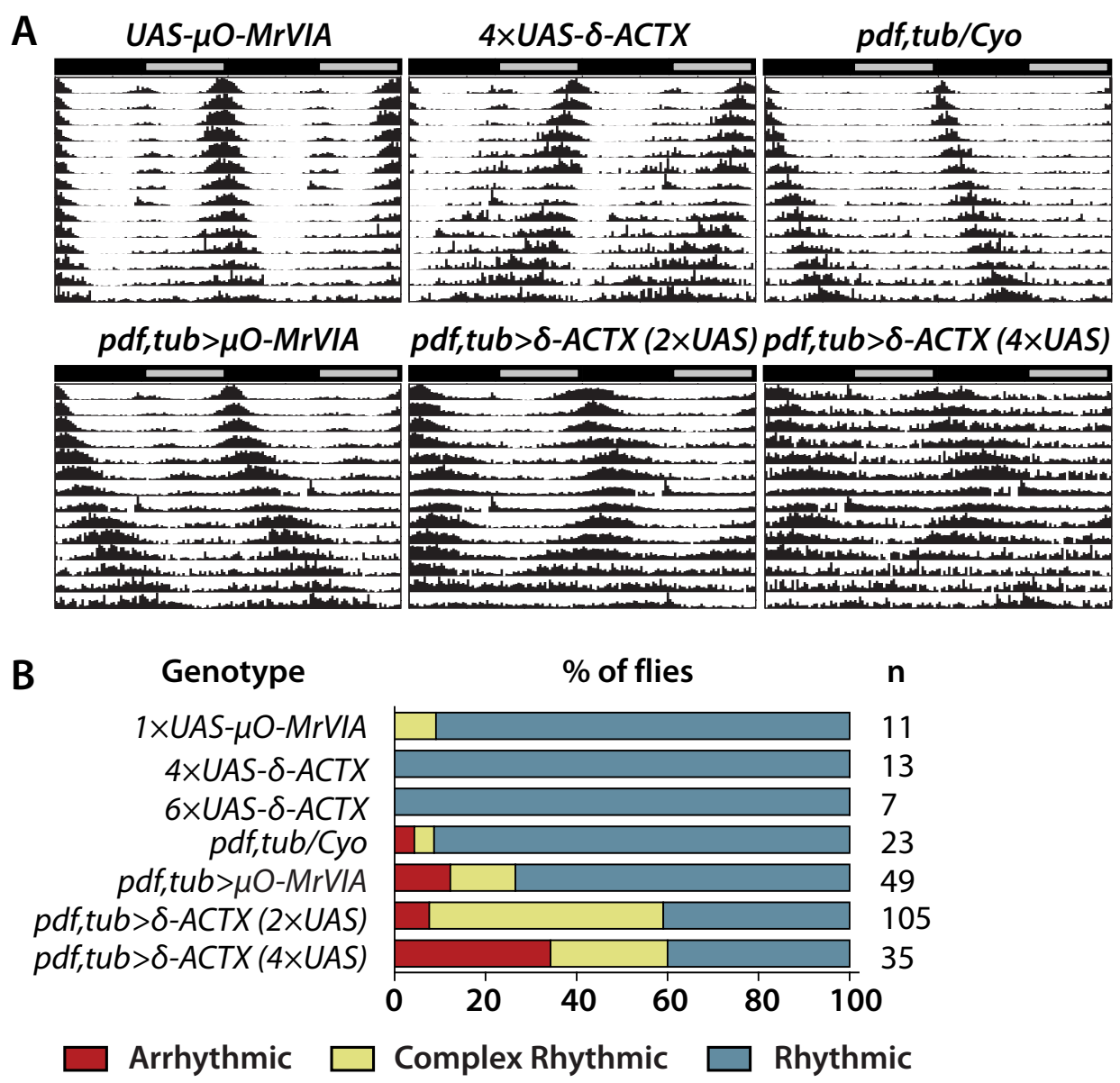
A
UAS-μO-MrVIA
4×UAS-δ-ACTX
pdf,tub/Cyo
pdf,tub>μO-MrVIA
pdf,tub>δ-ACTX (2×UAS)
pdf,tub>δ-ACTX (4×UAS)
B
Genotype
% of flies
n
1×UAS-μO-MrVIA
11
4×UAS-δ-ACTX
13
6×UAS-δ-ACTX
7
pdf,tub/Cyo
23
pdf,tub>μO-MrVIA
49
pdf,tub>δ-ACTX (2×UAS)
105
pdf,tub>δ-ACTX (4×UAS)
35
0
20
40
60
80
100
Arrhythmic
Complex Rhythmic
Rhythmic
