# Supplementary material for: Phase Coupling of a Circadian Neuropeptide With Rest/Activity Rhythms Detected Using a Membrane-Tethered Spider Toxin
Source: PLoS Biol. 2008 Nov 4;6(11):e273. doi: 10.1371/journal.pbio.0060273 (PMC2577701; doi:10.1371/journal.pbio.0060273)
Supplement: Supplementary file 4 [file pbio.0060273.sg004.pdf]

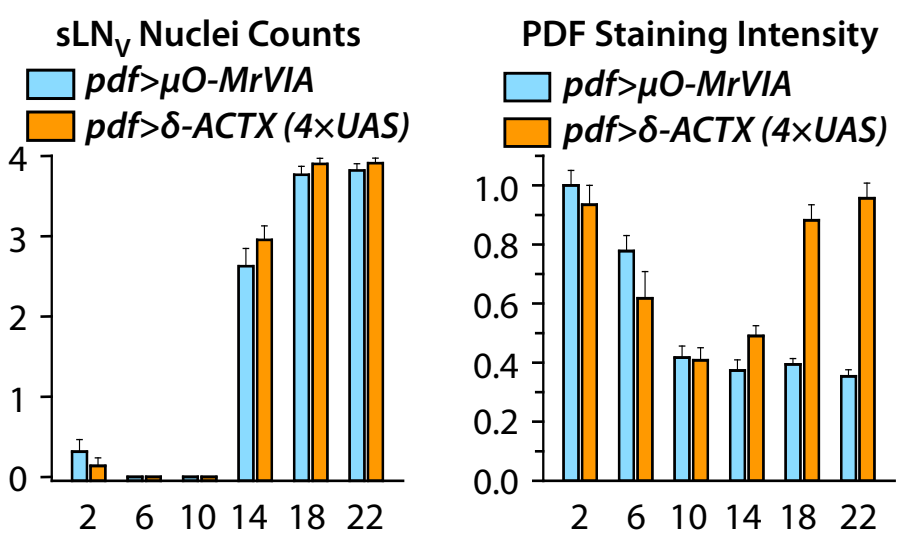

sLN$_V$ Nuclei Counts
pdf>μO-MrVIA
pdf>δ-ACTX (4×UAS)
0
1
2
3
4
2
6
10
14
18
22
PDF Staining Intensity
pdf>μO-MrVIA
pdf>δ-ACTX (4×UAS)
0.0
0.2
0.4
0.6
0.8
1.0
2
6
10
14
18
22
