# Supplementary material for: Phase Coupling of a Circadian Neuropeptide With Rest/Activity Rhythms Detected Using a Membrane-Tethered Spider Toxin
Source: PLoS Biol. 2008 Nov 4;6(11):e273. doi: 10.1371/journal.pbio.0060273 (PMC2577701; doi:10.1371/journal.pbio.0060273)
Supplement: Supplementary file 5 [file pbio.0060273.st001.doc]

Supplemental Table 1. Period and power of rhythmicity in different genotype of flies

| **Genotype** | **Arrhythmic** | **Complex Rhythmic** | | **Rhythmic** | | | **N** |
| --- | --- | --- | --- | --- | --- | --- | --- |
|  | **%** | **%** | **Period±SEM (hrs)** | **%** | **Period****±SEM (hrs)** | **Power±SEM** |  |
| UAS-µO-MrVIA | 5 | 3 |  | 92 | 23.7±0.06 | 84.8±6.41 | 37 |
| UAS- δ-ACTX-Hv1a (2×UAS) | 2 | 7 |  | 91 | 23.4±0.04 | 46.4±3.76 | 44 |
| UAS- δ-ACTX-Hv1a (4×UAS) | 3 | 0 |  | 97 | 23.4±0.03 | 46.0±4.38 | 33 |
| UAS- δ-ACTX-Hv1a (6×UAS) | 0 | 13 |  | 87 | 23.5±0.07 | 45.5±5.37 | 15 |
| pdf/Cyo | 0 | 5 |  | 95 | 24.1±0.04 | 94.4±7.68 | 56 |
| pdf/µO-MrVIA | 6 | 1 |  | 93 | 24.4±0.02 | 40.4±1.24 | 298 |
| pdf/δ-ACTX-Hv1a (1×UAS) | 30 | 32 | 25.0±0.04 22.1±0.05 | 38 | 24.5±0.09 | 22.0±1.12 | 523 |
| pdf/δ-ACTX-Hv1a (2×UAS) | 37 | 30 | 25.1±0.05 22.0±0.06 | 33 | 24.2±0.11 | 16.0±0.51 | 576 |
| pdf/δ-ACTX-Hv1a (4×UAS) | 55 | 13 | 25.5±0.10 22.5±0.10 | 32 | 24.7±0.15 | 15.3±0.66 | 247 |
| pdf/δ-ACTX-Hv1a (6×UAS) | 42 | 23 | 25.2±0.09 21.4±0.14 | 35 | 24.4±0.25 | 20.3±1.94 | 128 |
| pdf/κ-ACTX-Hv1c (7a) | 16 | 54 | 24.3±0.07 21.9±0.05 | 30 | 24.3±0.09 | 26.9±7.17 | 37 |
| pdf/κ-ACTX-Hv1c (12b) | 7 | 48 | 24.5±0.05 22.1±0.04 | 45 | 24.3±0.11 | 30.9±3.45 | 73 |
| pdf/PLTXII (1a) | 10 | 0 |  | 90 | 24.4±0.04 | 70.2±6.54 | 31 |
| pdf/PLTXII (3a) | 3 | 0 |  | 97 | 24.6±0.05 | 40.6±3.34 | 31 |
| pdf,tub/Cyo | 13 | 0 |  | 87 | 24.7±0.06 | 32.4±3.73 | 23 |
| pdf,tub/µO-MrVIA | 6 | 0 |  | 94 | 24.6±0.05 | 37.6±2.95 | 32 |
| pdf,tub/ω-ACTX-Hv1c (1a) | 39 | 39 | 24.4±0.40 22.5±0.37 | 22 | 23.8±0.50 | 11.9±0.77 | 36 |
| pdf,tub/ω-ACTX-Hv1c (2a) | 48 | 10 | 25.1±0.10 21.8±0.10 | 42 | 22.8±0.47 | 15.5±1.74 | 31 |
